# Supplementary material for: Phylogeny-driven design of broadly protective sarbecovirus receptor-binding domain nanoparticle vaccines
Source: bioRxiv. 2025 May 13:2025.05.11.652904. Preprint. [Version 1] doi: 10.1101/2025.05.11.652904 (PMC12132498; doi:10.1101/2025.05.11.652904)
Supplement: Supplement 1 [file NIHPP2025.05.11.652904v1-supplement-1.pdf]

bioRxiv preprint doi: https://doi.org/10.1101/2025.05.11.652904; this version posted May 13, 2025. The copyright holder for this preprint (which was not certified by peer review) is the author/funder, who has granted bioRxiv a license to display the preprint in perpetuity. It is made available under aCC-BY 4.0 International license.

Supplemental Figures

A SARS-CoV-2 Wu-G614 VSV

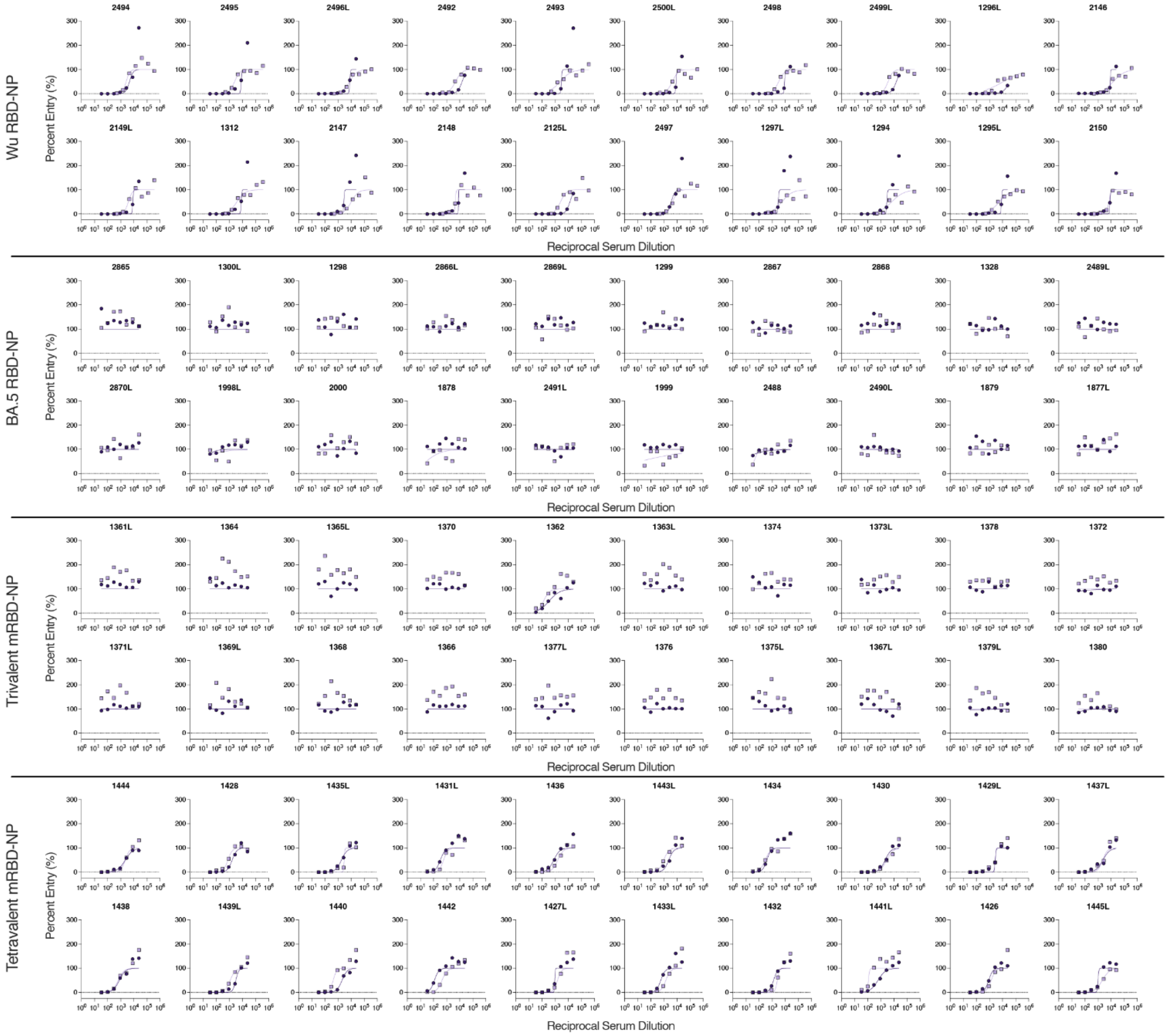

bioRxiv preprint doi: https://doi.org/10.1101/2025.05.11.652904; this version posted May 13, 2025. The copyright holder for this preprint (which was not certified by peer review) is the author/funder, who has granted bioRxiv a license to display the preprint in perpetuity. It is made available under aCC-BY 4.0 International license.

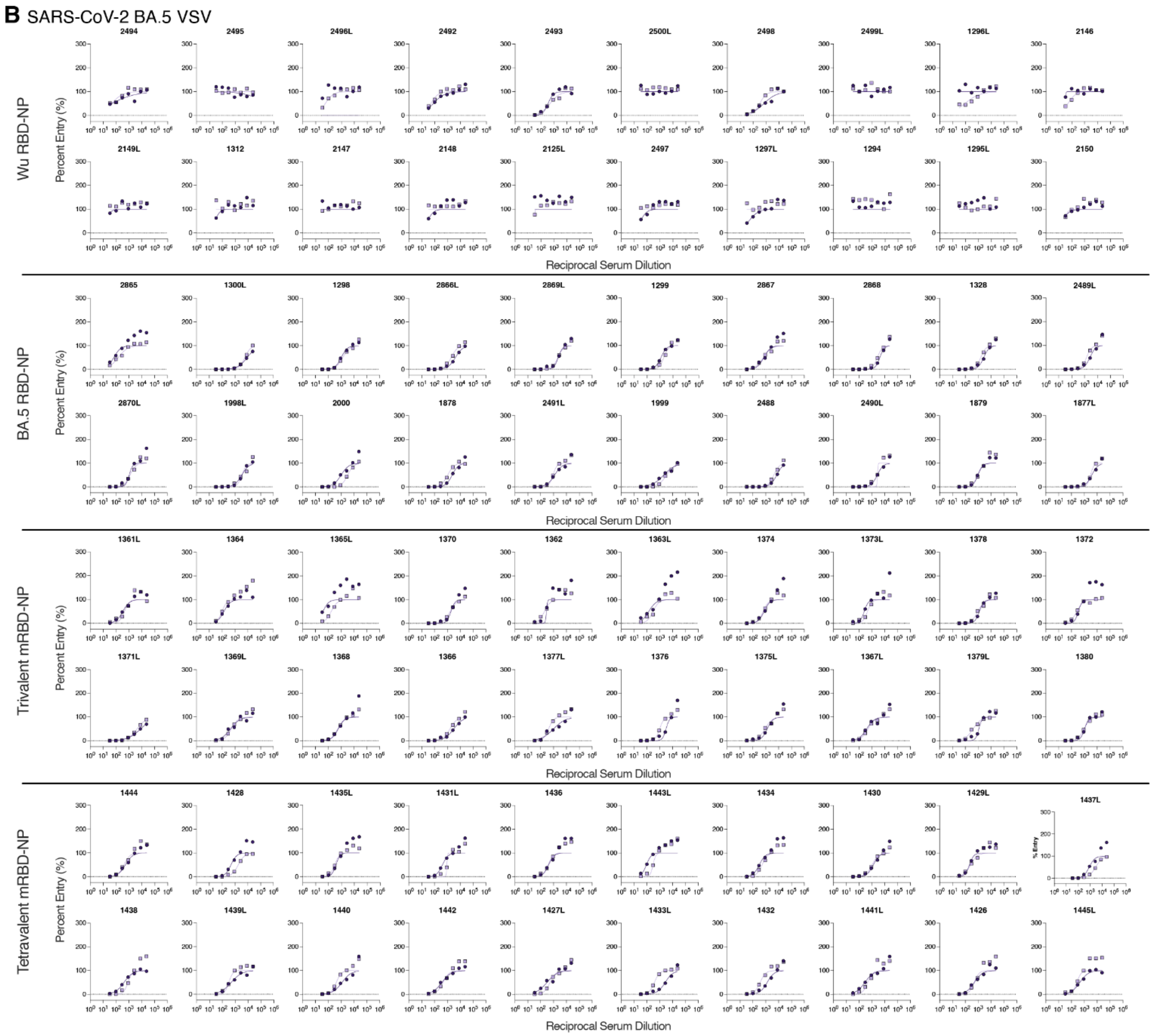

bioRxiv preprint doi: https://doi.org/10.1101/2025.05.11.652904; this version posted May 13, 2025. The copyright holder for this preprint (which was not certified by peer review) is the author/funder, who has granted bioRxiv a license to display the preprint in perpetuity. It is made available under aCC-BY 4.0 International license.

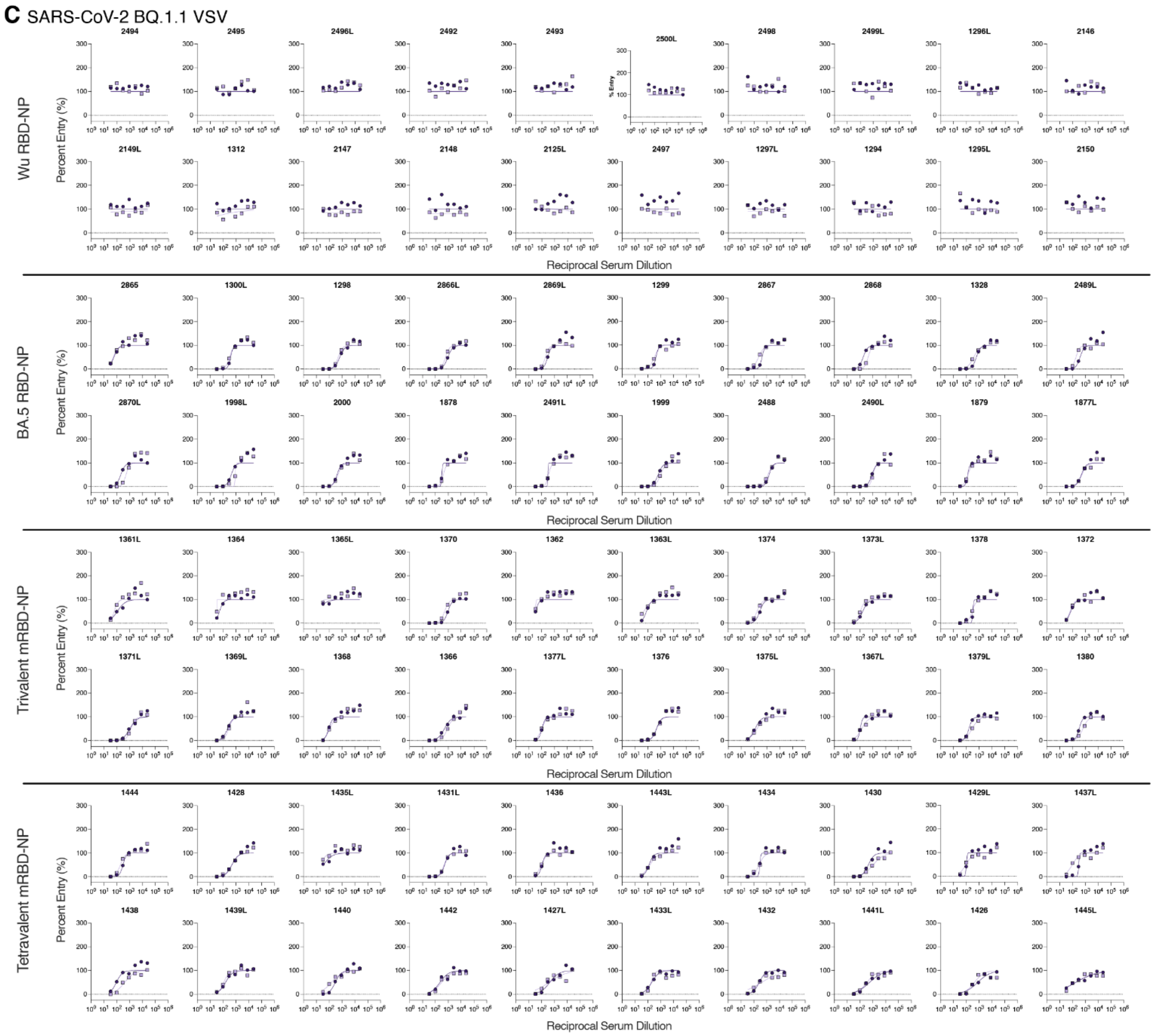

bioRxiv preprint doi: https://doi.org/10.1101/2025.05.11.652904; this version posted May 13, 2025. The copyright holder for this preprint (which was not certified by peer review) is the author/funder, who has granted bioRxiv a license to display the preprint in perpetuity. It is made available under aCC-BY 4.0 International license.

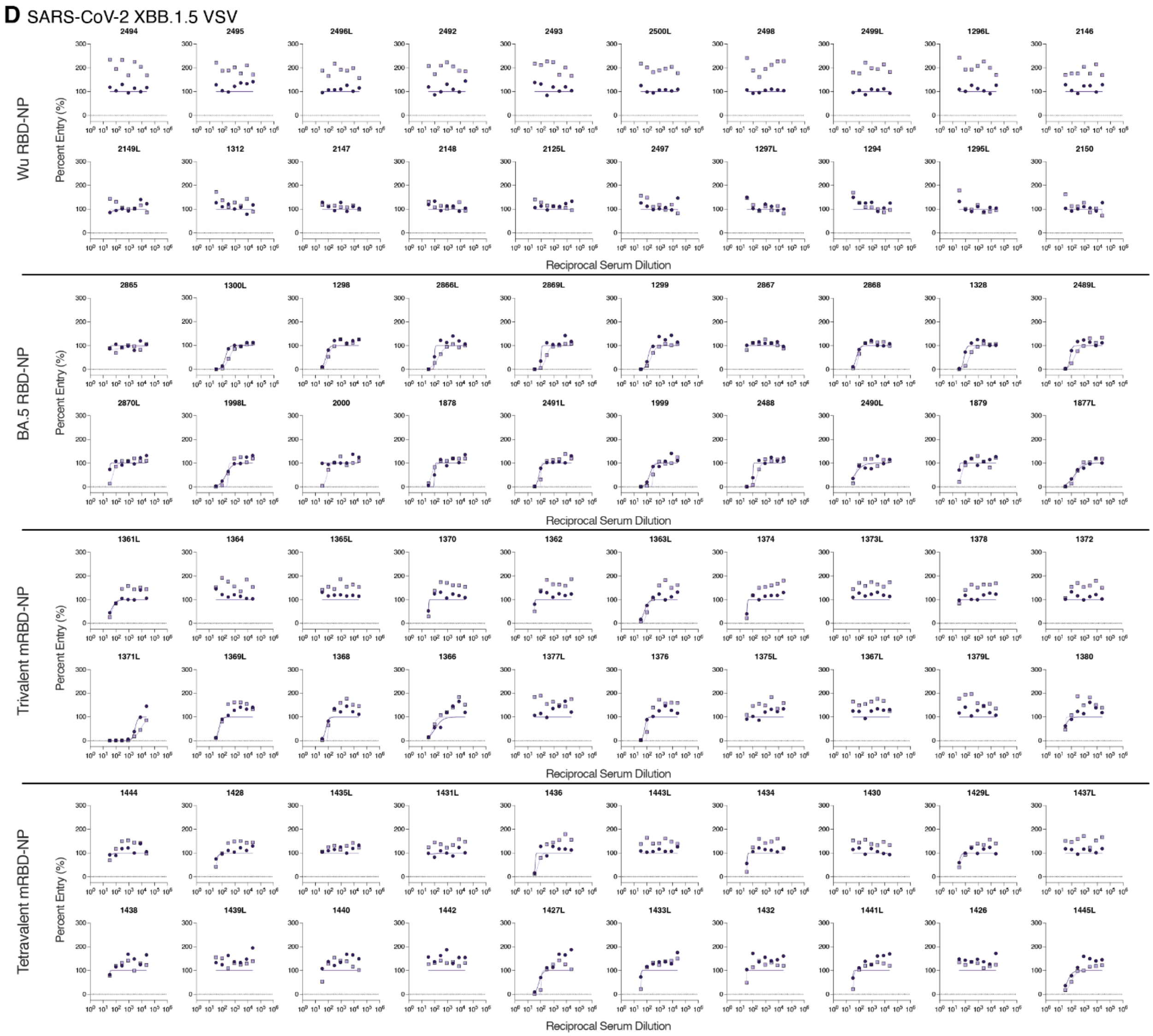

bioRxiv preprint doi: https://doi.org/10.1101/2025.05.11.652904; this version posted May 13, 2025. The copyright holder for this preprint (which was not certified by peer review) is the author/funder, who has granted bioRxiv a license to display the preprint in perpetuity. It is made available under aCC-BY 4.0 International license.

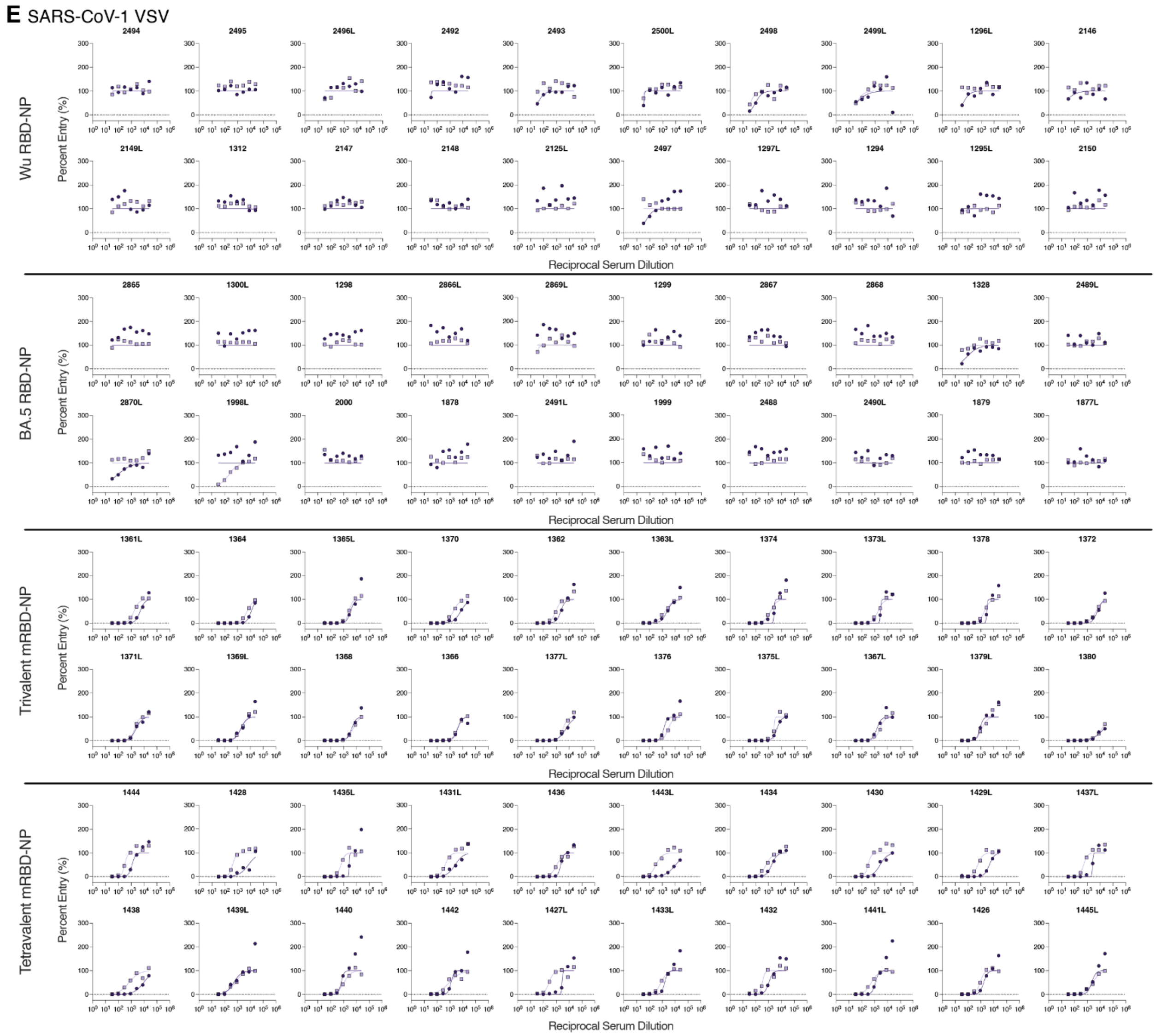

bioRxiv preprint doi: https://doi.org/10.1101/2025.05.11.652904; this version posted May 13, 2025. The copyright holder for this preprint (which was not certified by peer review) is the author/funder, who has granted bioRxiv a license to display the preprint in perpetuity. It is made available under aCC-BY 4.0 International license.

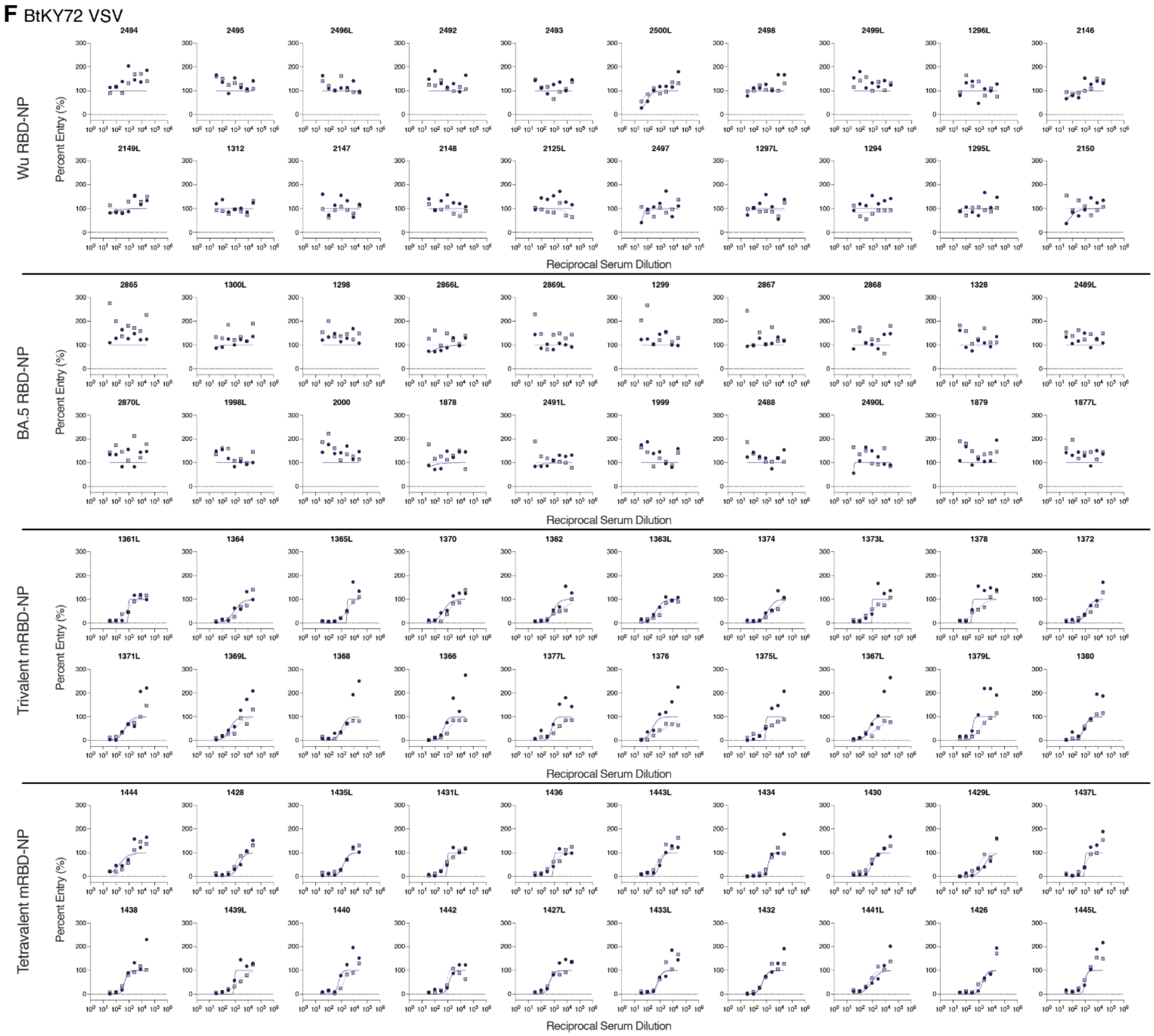

bioRxiv preprint doi: https://doi.org/10.1101/2025.05.11.652904; this version posted May 13, 2025. The copyright holder for this preprint (which was not certified by peer review) is the author/funder, who has granted bioRxiv a license to display the preprint in perpetuity. It is made available under aCC-BY 4.0 International license.

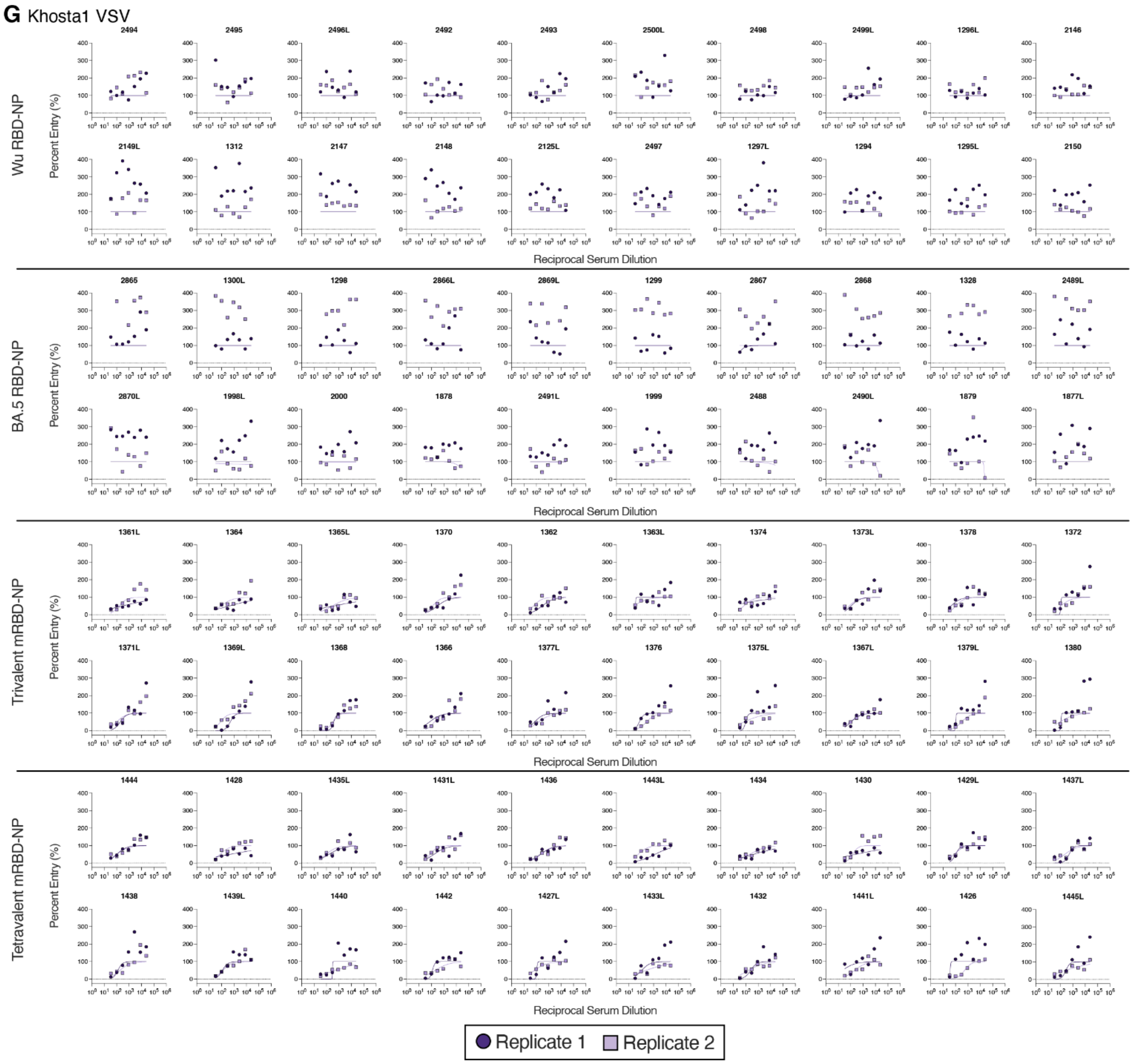

Figure S1. Neutralization dose-response curves for RBD-NP-immunized naive mice. Serum neutralizing antibody titers were assessed using VSV pseudotyped with the **A)** SARS-CoV-2 Wu-G614, **B)** SARS-CoV-2 BA.5, **C)** SARS-CoV-2 BQ.1.1, **D)** SARS-CoV-2 XBB.1.5, **E)** SARS-CoV-1, **F)** BtKY72, or **G)** Khosta1 S. Two biological replicates were conducted using distinct batches of pseudovirus.

bioRxiv preprint doi: https://doi.org/10.1101/2025.05.11.652904; this version posted May 13, 2025. The copyright holder for this preprint (which was not certified by peer review) is the author/funder, who has granted bioRxiv a license to display the preprint in perpetuity. It is made available under aCC-BY 4.0 International license.

A SARS-CoV-2 Wu-G614 VSV

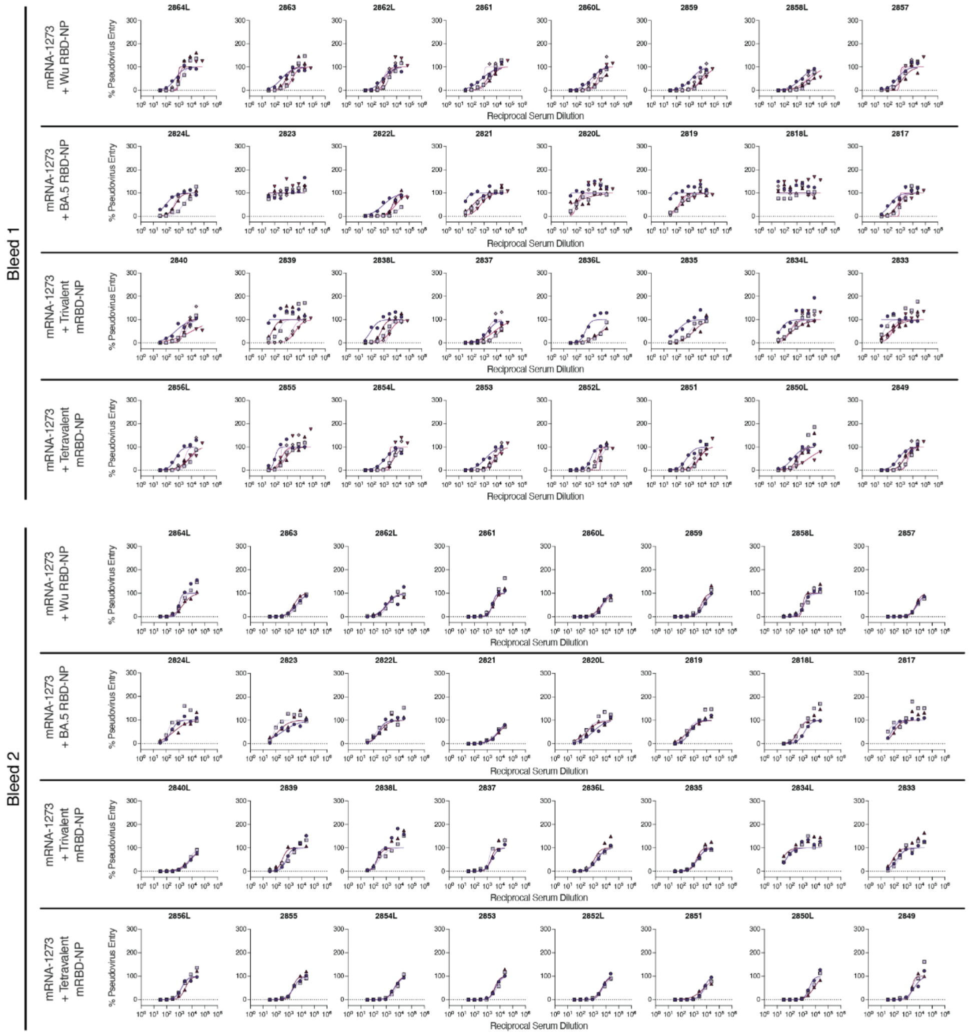

bioRxiv preprint doi: https://doi.org/10.1101/2025.05.11.652904; this version posted May 13, 2025. The copyright holder for this preprint (which was not certified by peer review) is the author/funder, who has granted bioRxiv a license to display the preprint in perpetuity. It is made available under aCC-BY 4.0 International license.

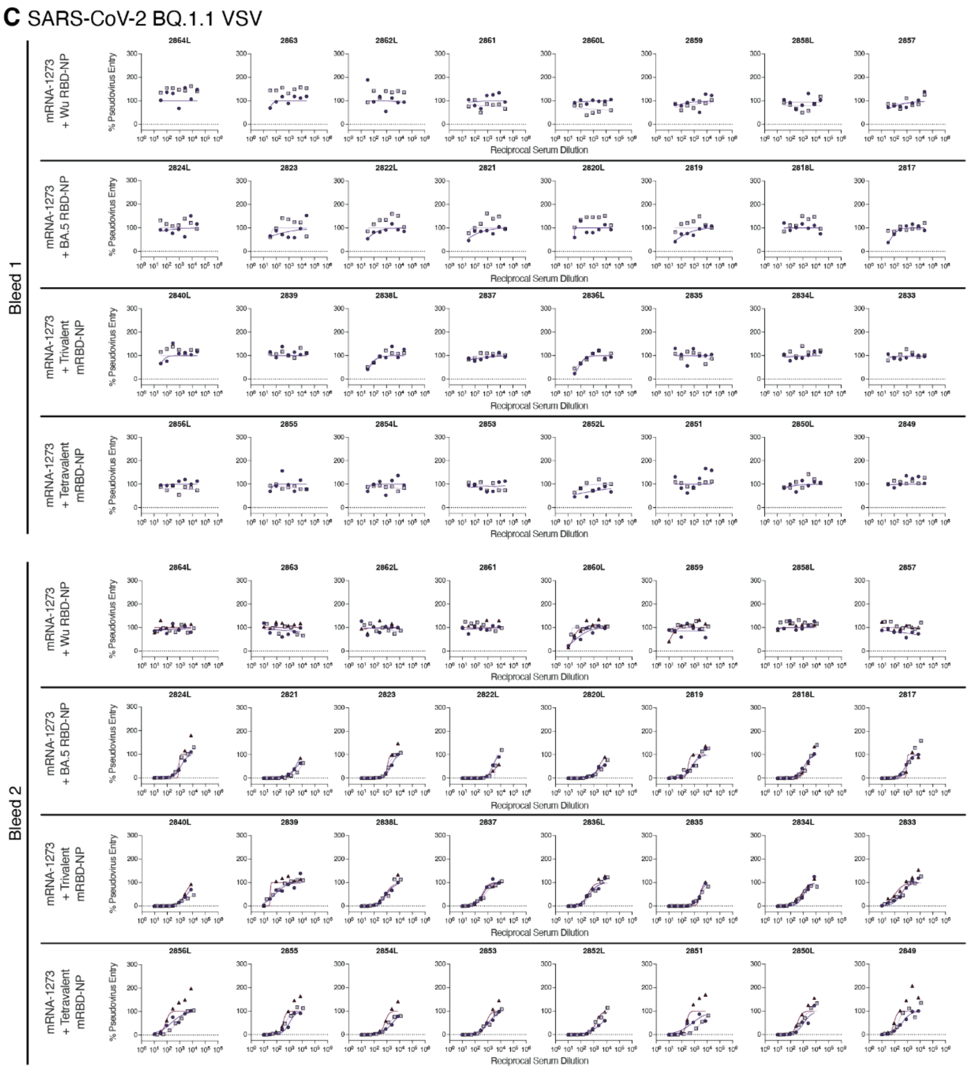

bioRxiv preprint doi: https://doi.org/10.1101/2025.05.11.652904; this version posted May 13, 2025. The copyright holder for this preprint (which was not certified by peer review) is the author/funder, who has granted bioRxiv a license to display the preprint in perpetuity. It is made available under aCC-BY 4.0 International license.

D SARS-CoV-2 XBB.1.5 VSV

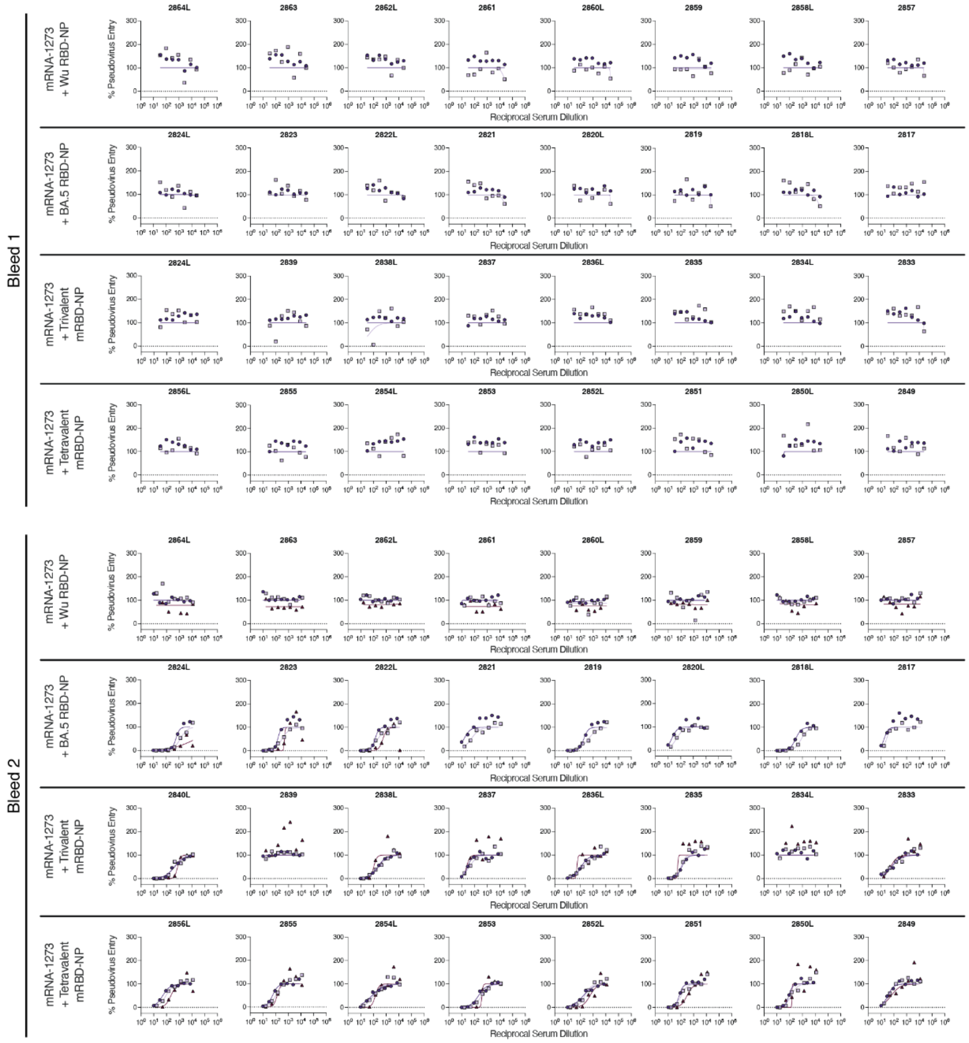

bioRxiv preprint doi: https://doi.org/10.1101/2025.05.11.652904; this version posted May 13, 2025. The copyright holder for this preprint (which was not certified by peer review) is the author/funder, who has granted bioRxiv a license to display the preprint in perpetuity. It is made available under aCC-BY 4.0 International license.

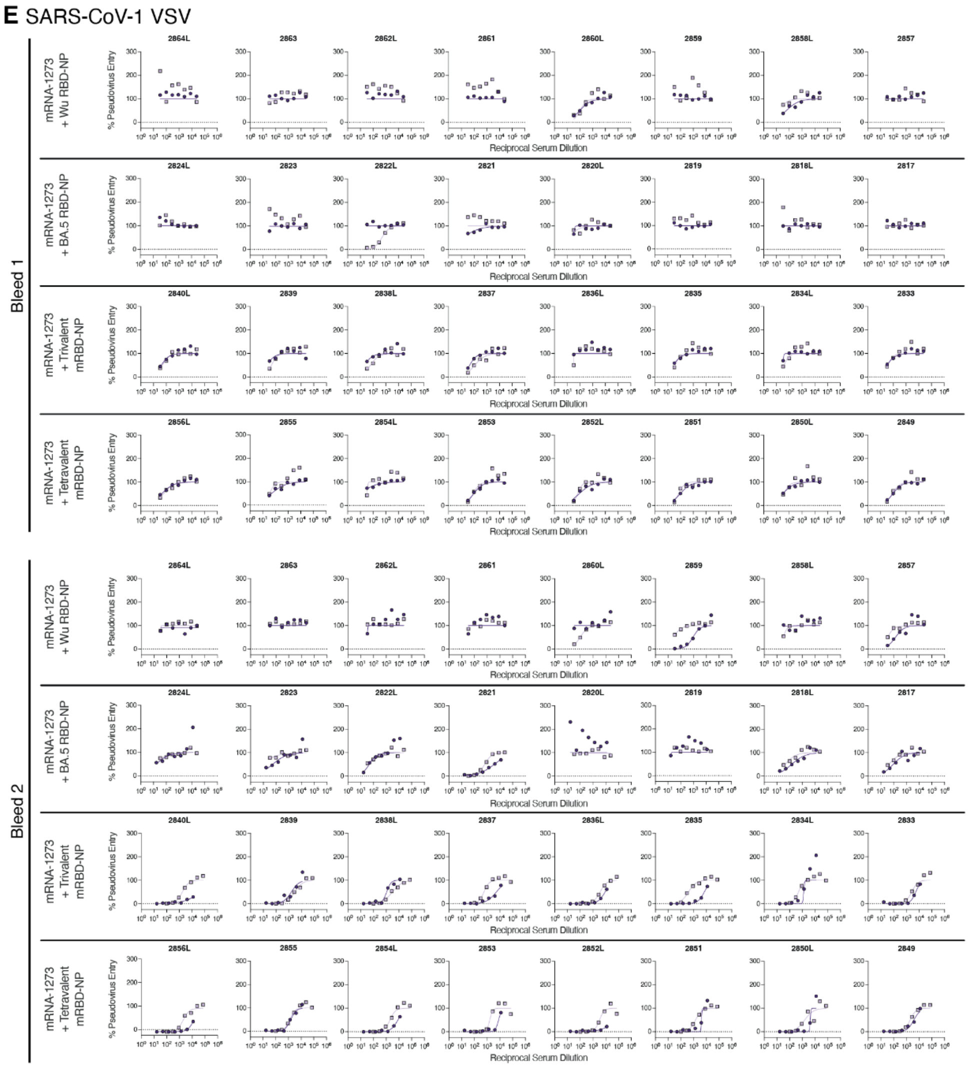

bioRxiv preprint doi: https://doi.org/10.1101/2025.05.11.652904; this version posted May 13, 2025. The copyright holder for this preprint (which was not certified by peer review) is the author/funder, who has granted bioRxiv a license to display the preprint in perpetuity. It is made available under aCC-BY 4.0 International license.

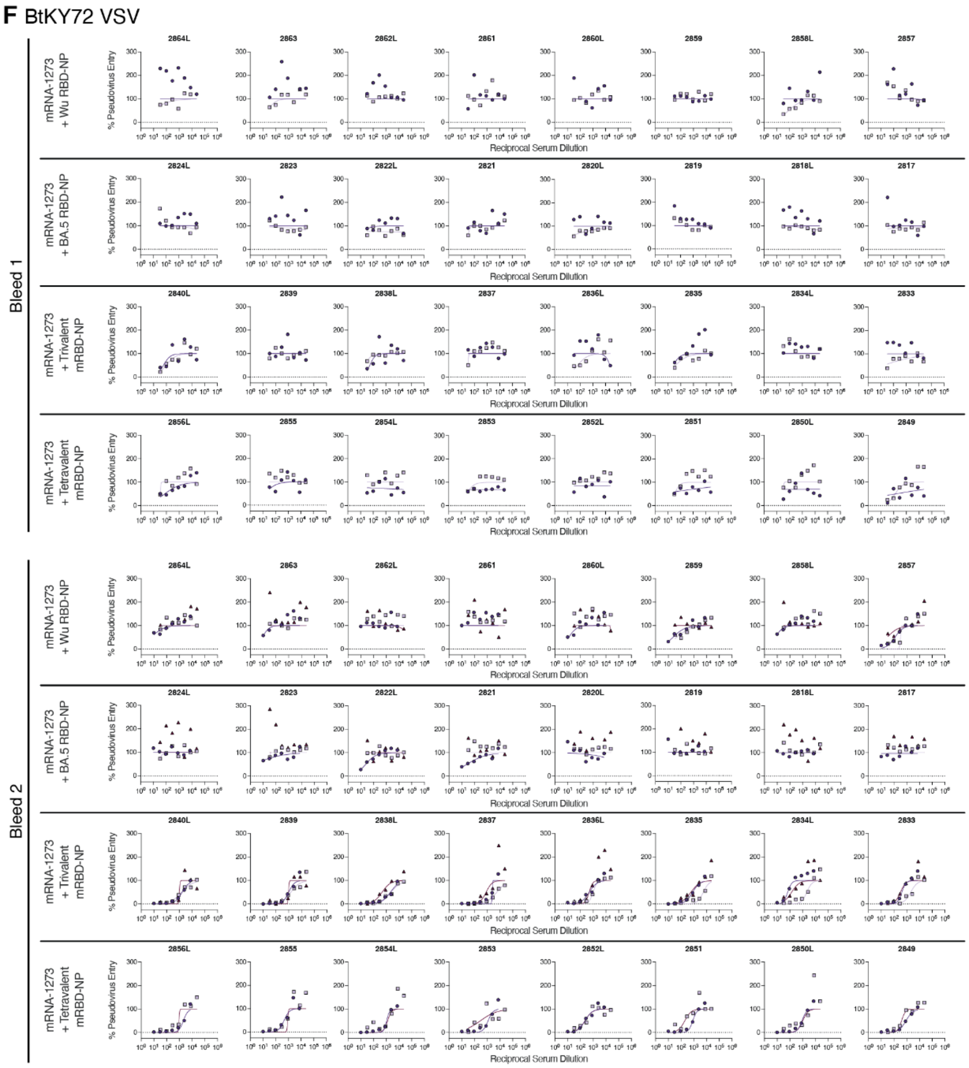

bioRxiv preprint doi: https://doi.org/10.1101/2025.05.11.652904; this version posted May 13, 2025. The copyright holder for this preprint (which was not certified by peer review) is the author/funder, who has granted bioRxiv a license to display the preprint in perpetuity. It is made available under aCC-BY 4.0 International license.

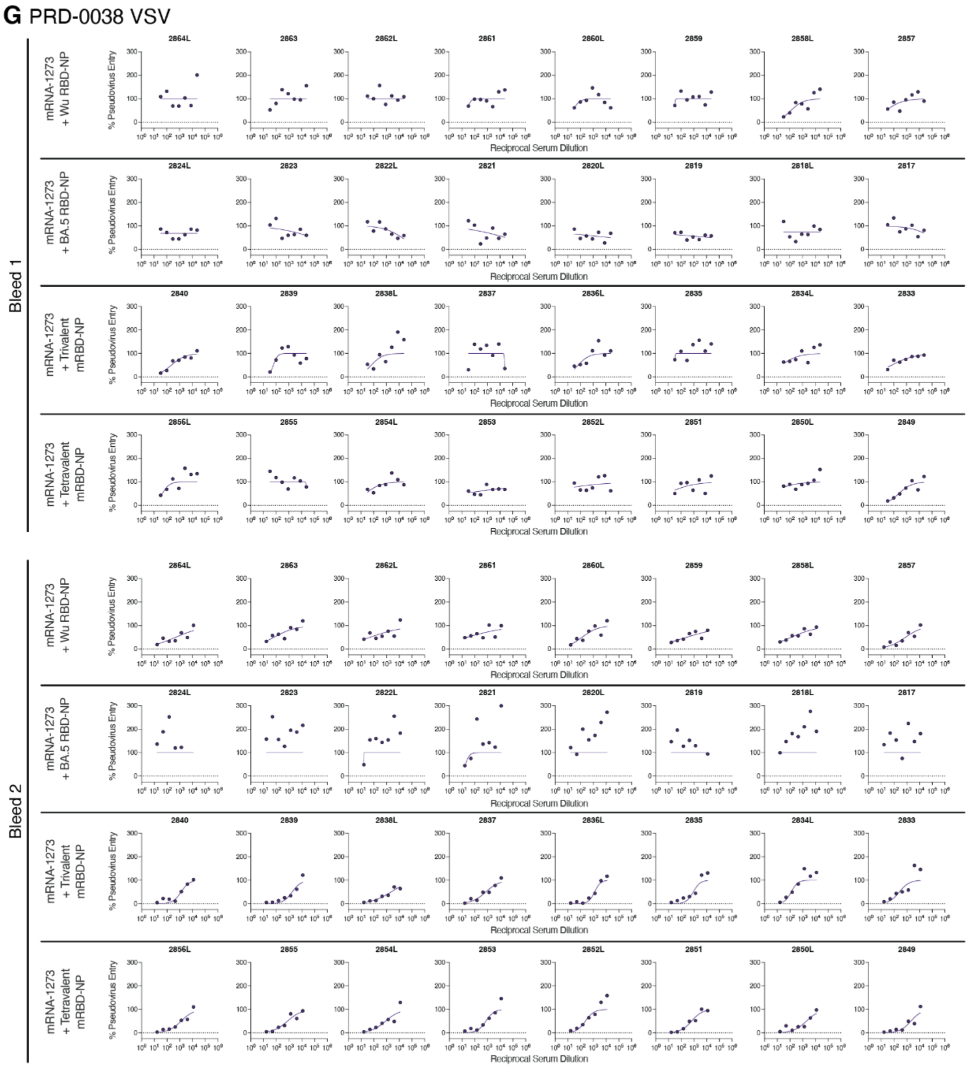

bioRxiv preprint doi: https://doi.org/10.1101/2025.05.11.652904; this version posted May 13, 2025. The copyright holder for this preprint (which was not certified by peer review) is the author/funder, who has granted bioRxiv a license to display the preprint in perpetuity. It is made available under aCC-BY 4.0 International license.

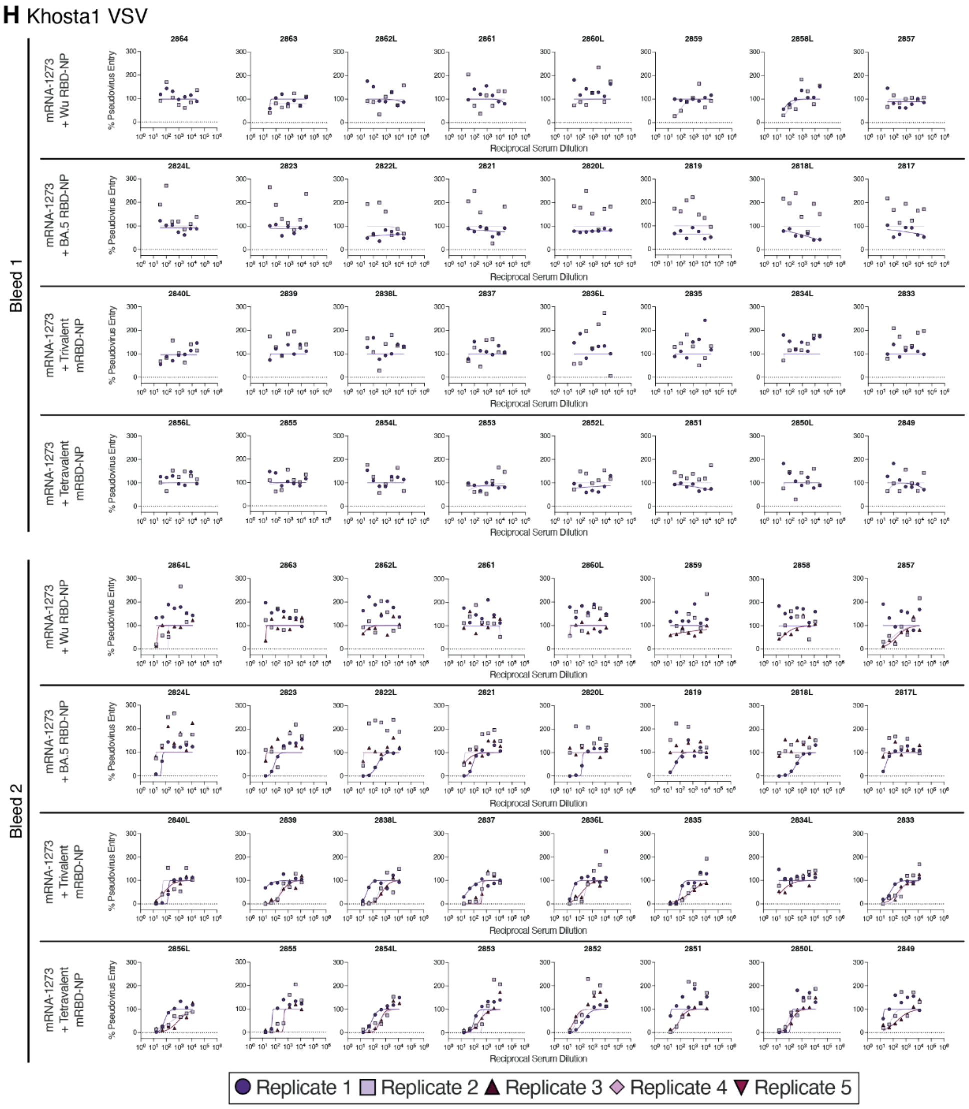

Figure S2. Neutralization dose-response curves for RBD-NP-immunized pre-immune mice. Serum neutralizing antibody titers were assessed using VSV pseudotyped with the **A)** SARS-CoV-2 Wu-G614, **B)** SARS-CoV-2 BA.5, **C)** SARS-CoV-2 BQ.1.1, **D)** SARS-CoV-2 XBB.1.5, **E)** SARS-CoV-1, **F)** BtKY72, **G)** PRD-0038, or **H)** Khosta1 S. One to five biological replicates were conducted using distinct batches of pseudovirus.

bioRxiv preprint doi: https://doi.org/10.1101/2025.05.11.652904; this version posted May 13, 2025. The copyright holder for this preprint (which was not certified by peer review) is the author/funder, who has granted bioRxiv a license to display the preprint in perpetuity. It is made available under aCC-BY 4.0 International license.

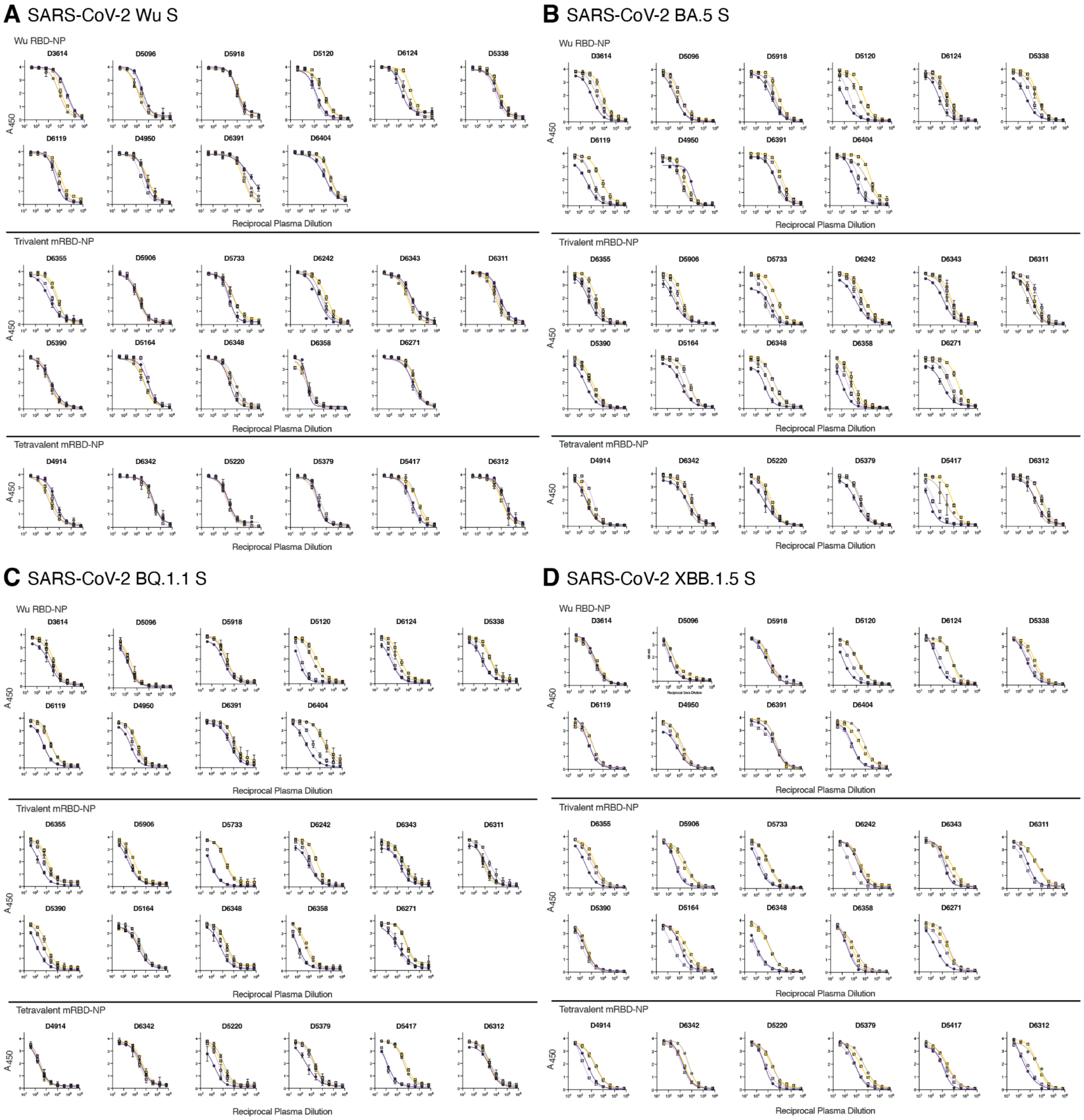

bioRxiv preprint doi: https://doi.org/10.1101/2025.05.11.652904; this version posted May 13, 2025. The copyright holder for this preprint (which was not certified by peer review) is the author/funder, who has granted bioRxiv a license to display the preprint in perpetuity. It is made available under aCC-BY 4.0 International license.

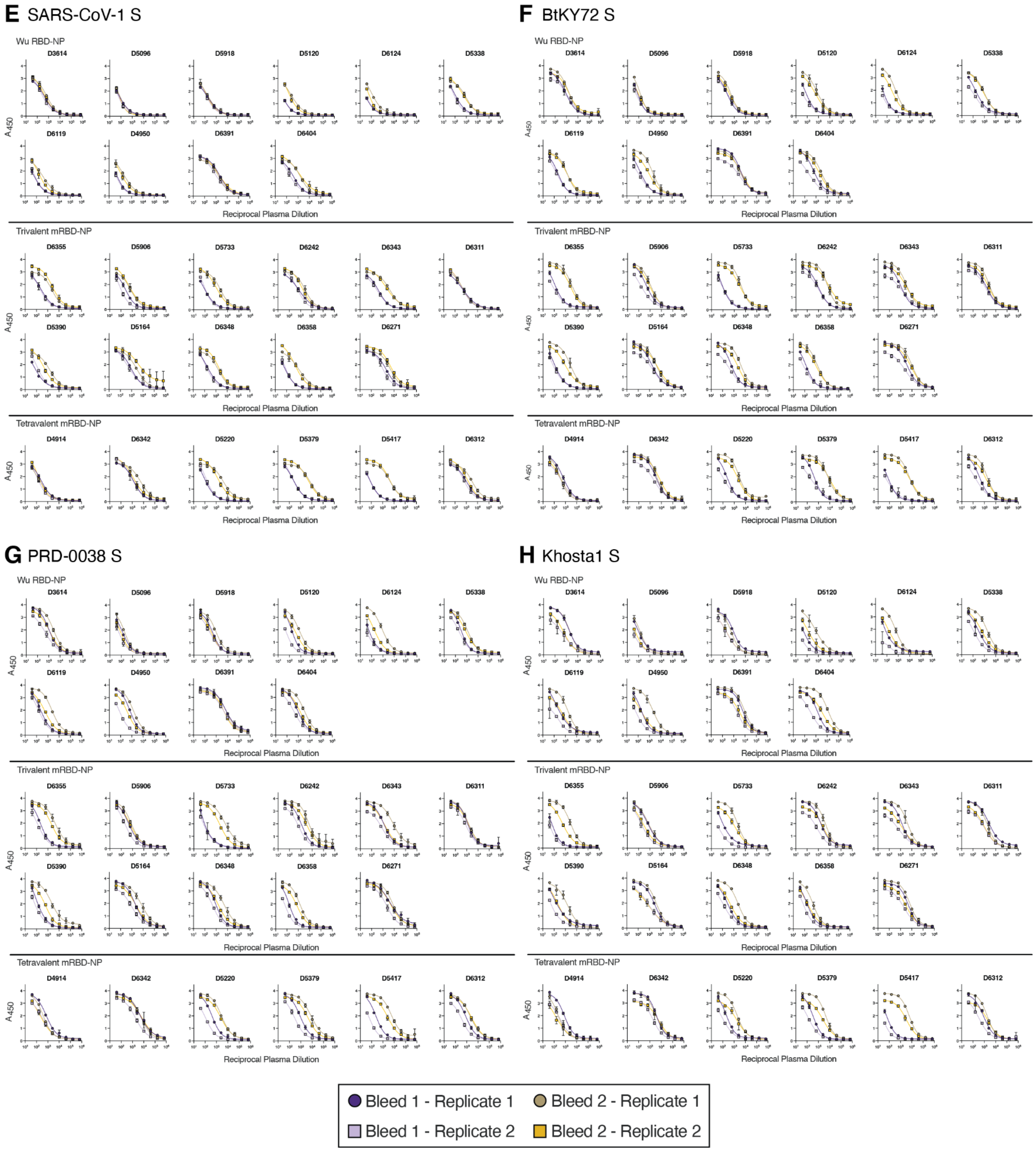

Figure S3. S glycoprotein ELISA dose-response curves for RBD-NP-immunized AGMs. Plasma binding antibody titers were assessed using the **A)** SARS-CoV-2 Wu, **B)** SARS-CoV-2 BA.5, **C)** SARS-CoV-2 BQ.1.1, **D)** SARS-CoV-2 XBB.1.5, **E)** SARS-CoV-1, **F)** BtKY72, **G)** PRD-0038, or **H)** Khosta1 S. Two biological replicates were each conducted in technical duplicates using distinct batches of proteins.

bioRxiv preprint doi: https://doi.org/10.1101/2025.05.11.652904; this version posted May 13, 2025. The copyright holder for this preprint (which was not certified by peer review) is the author/funder, who has granted bioRxiv a license to display the preprint in perpetuity. It is made available under aCC-BY 4.0 International license.

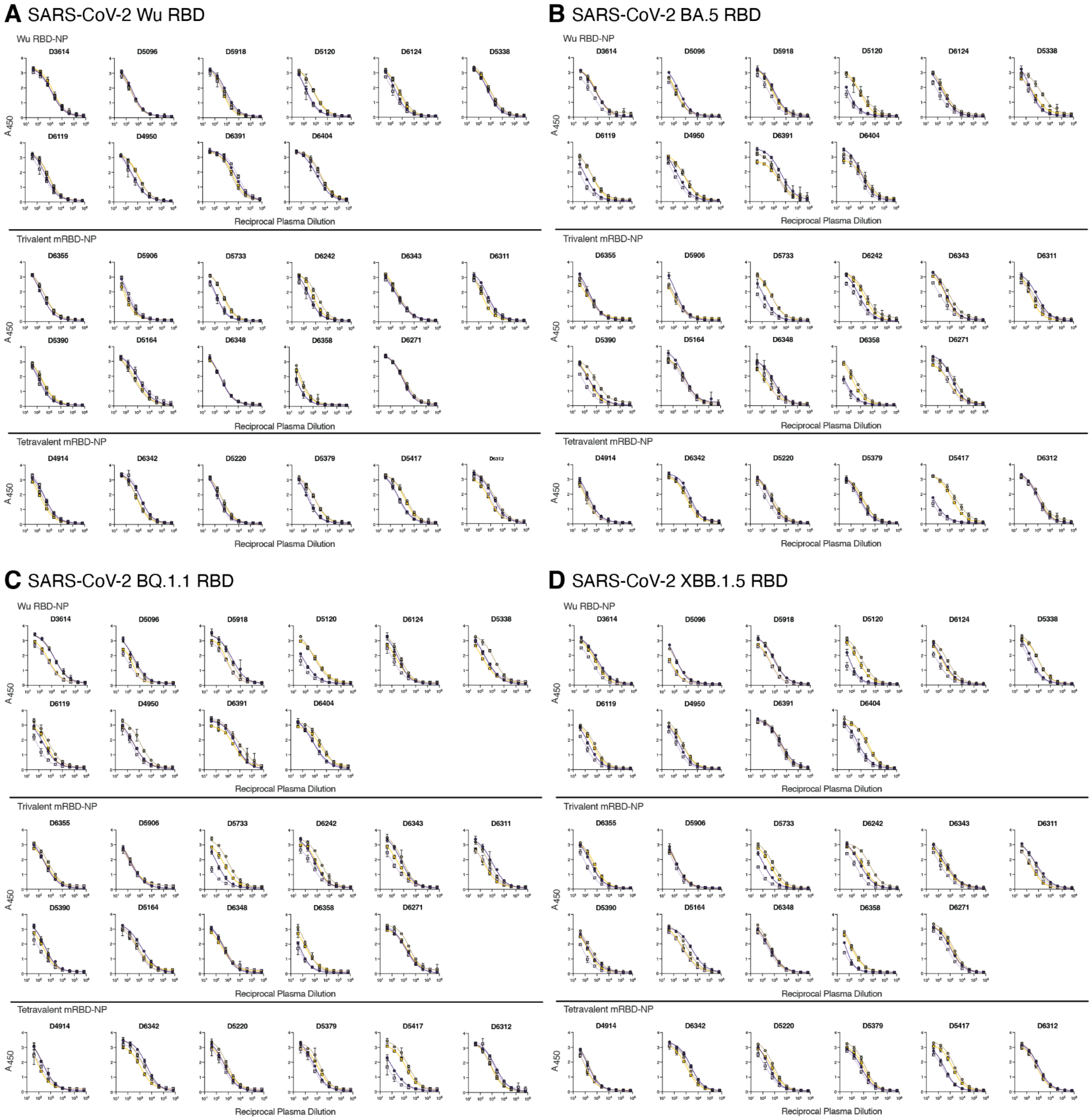

bioRxiv preprint doi: https://doi.org/10.1101/2025.05.11.652904; this version posted May 13, 2025. The copyright holder for this preprint (which was not certified by peer review) is the author/funder, who has granted bioRxiv a license to display the preprint in perpetuity. It is made available under aCC-BY 4.0 International license.

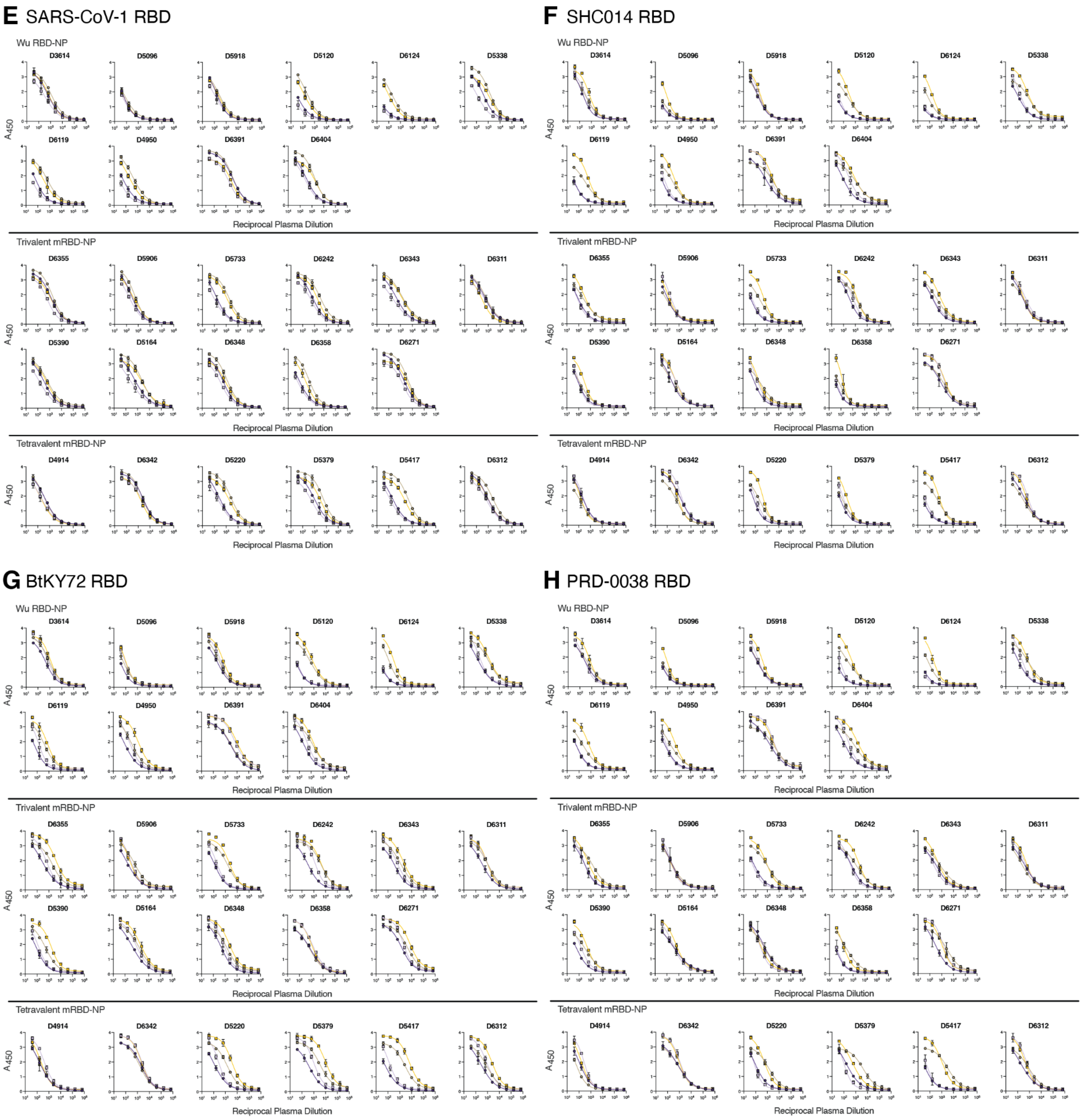

bioRxiv preprint doi: https://doi.org/10.1101/2025.05.11.652904; this version posted May 13, 2025. The copyright holder for this preprint (which was not certified by peer review) is the author/funder, who has granted bioRxiv a license to display the preprint in perpetuity. It is made available under aCC-BY 4.0 International license.

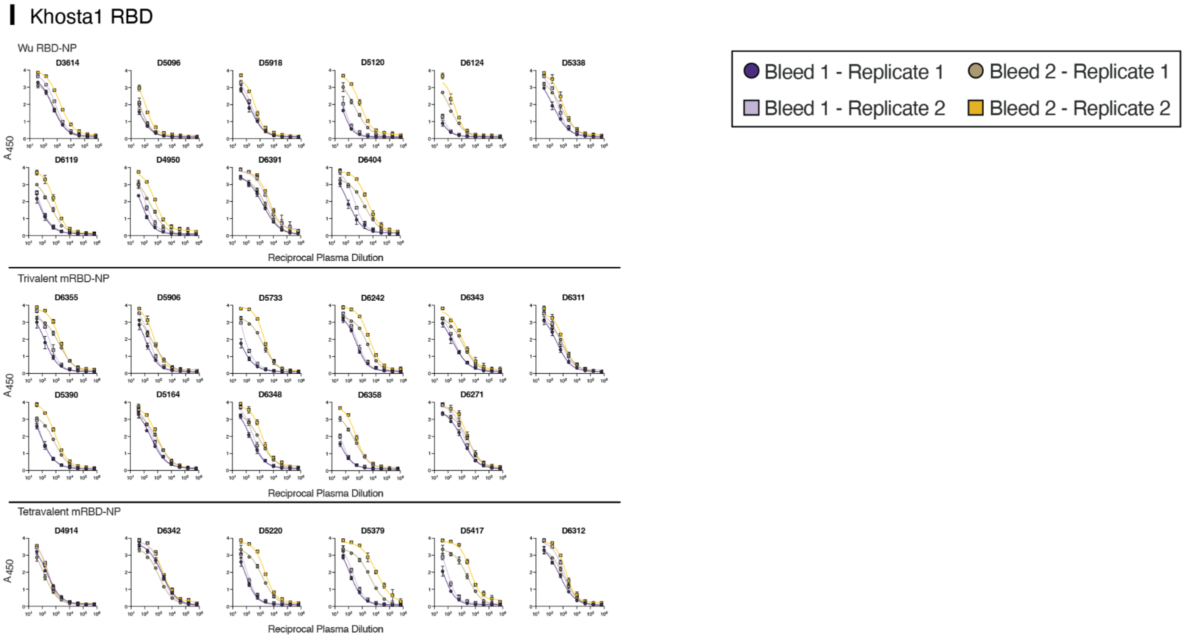

Figure S4. RBD ELISA dose-response curves for RBD-NP-immunized AGMs. Plasma binding antibody titers were assessed using the **A)** SARS-CoV-2 Wu, **B)** SARS-CoV-2 BA.5, **C)** SARS-CoV-2 BQ.1.1, **D)** SARS-CoV-2 XBB.1.5, **E)** SARS-CoV-1, **F)** RsSHC014, **G)** BtKY72, **H)** PRD-0038, or **I)** Khosta1 RBD. Two biological replicates were each conducted in technical duplicates using distinct batches of proteins.

bioRxiv preprint doi: https://doi.org/10.1101/2025.05.11.652904; this version posted May 13, 2025. The copyright holder for this preprint (which was not certified by peer review) is the author/funder, who has granted bioRxiv a license to display the preprint in perpetuity. It is made available under aCC-BY 4.0 International license.

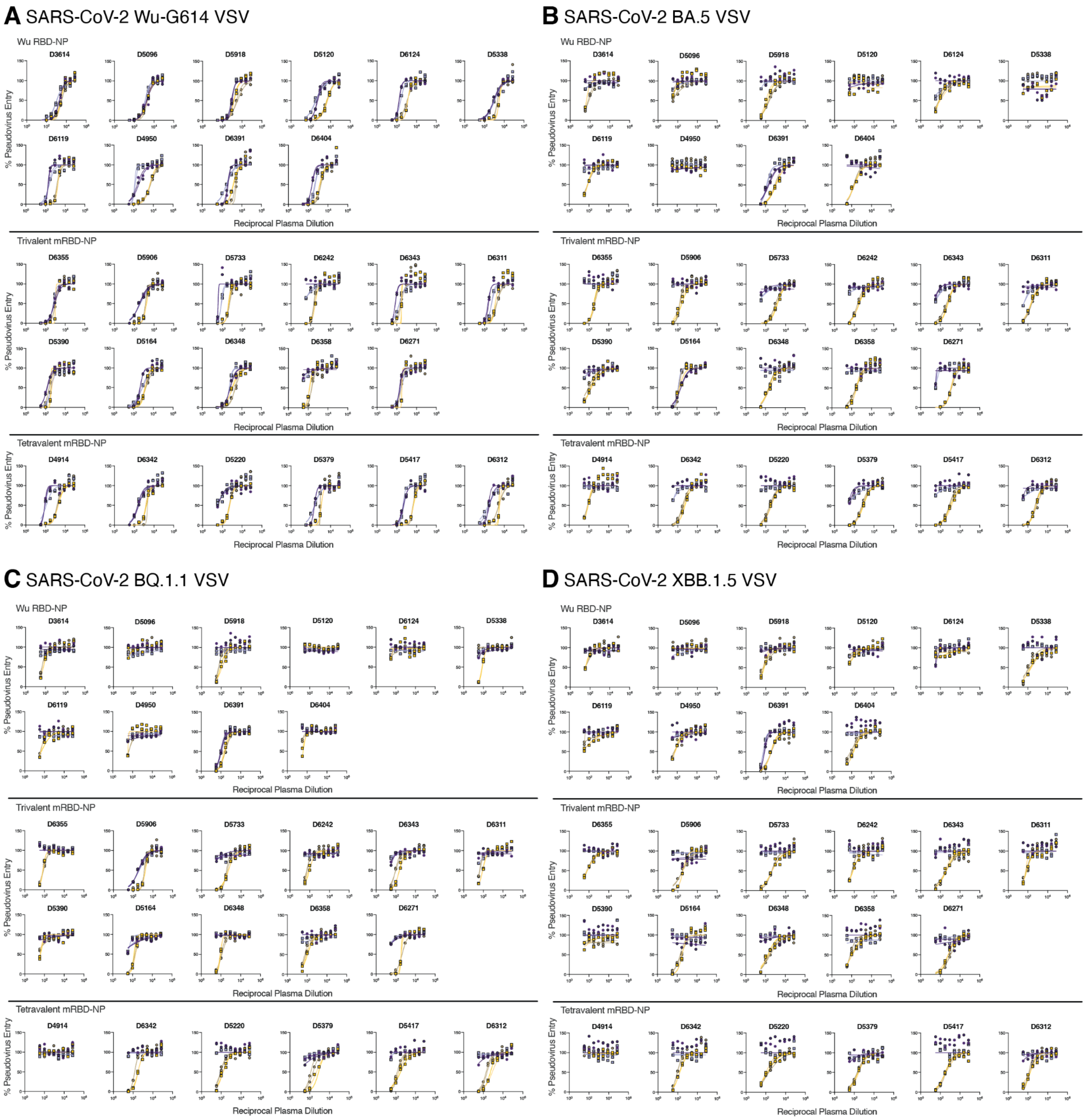

bioRxiv preprint doi: https://doi.org/10.1101/2025.05.11.652904; this version posted May 13, 2025. The copyright holder for this preprint (which was not certified by peer review) is the author/funder, who has granted bioRxiv a license to display the preprint in perpetuity. It is made available under aCC-BY 4.0 International license.

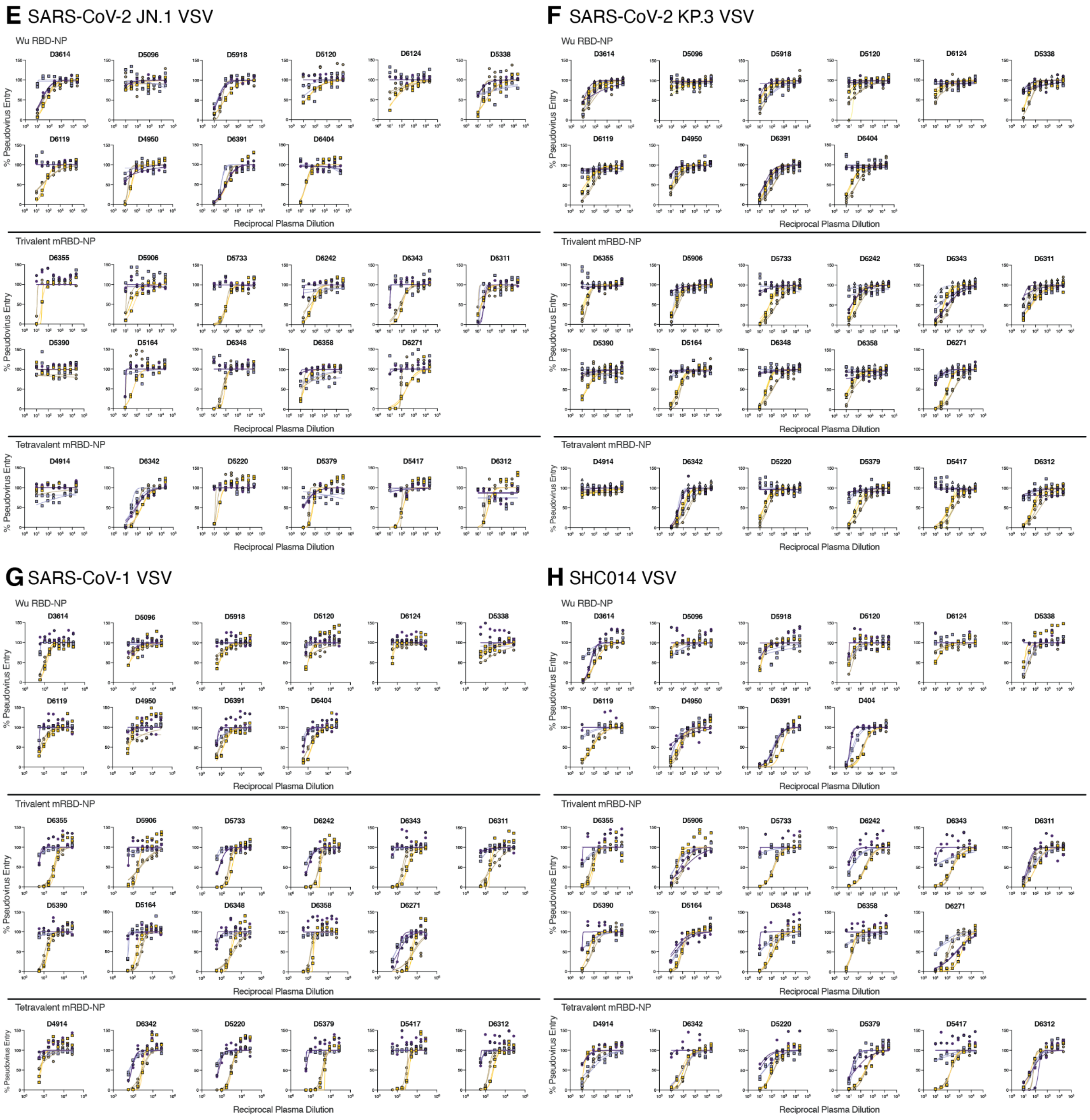

bioRxiv preprint doi: https://doi.org/10.1101/2025.05.11.652904; this version posted May 13, 2025. The copyright holder for this preprint (which was not certified by peer review) is the author/funder, who has granted bioRxiv a license to display the preprint in perpetuity. It is made available under aCC-BY 4.0 International license.

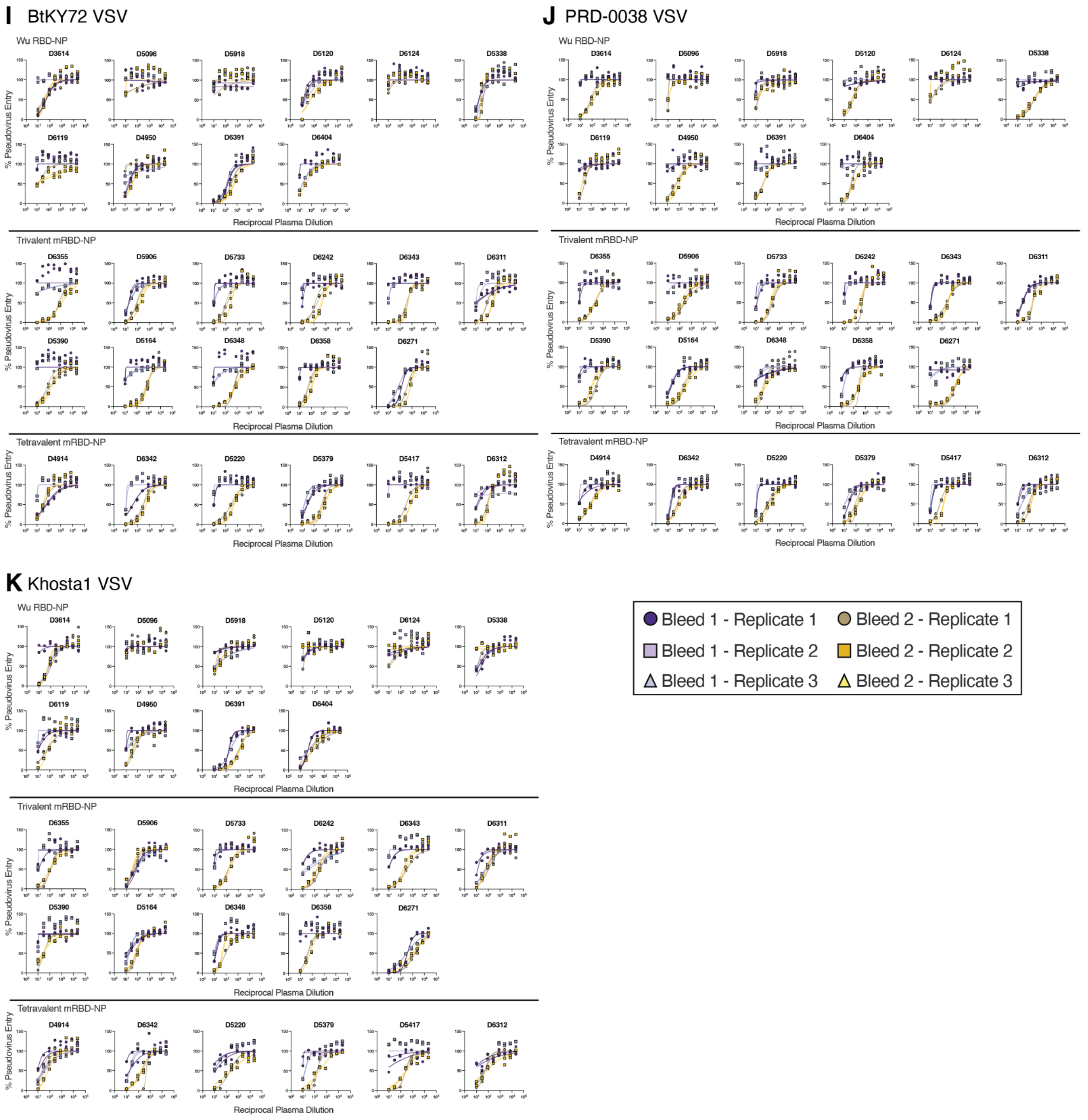

Figure S5. Neutralization dose-response curves for RBD-NP-immunized AGMs. Plasma neutralizing antibody titers were assessed using VSV pseudotyped with the **A)** SARS-CoV-2 Wu-G614, **B)** SARS-CoV-2 BA.5, **C)** SARS-CoV-2 BQ.1.1, **D)** SARS-CoV-2 XBB.1.5, **E)** SARS-CoV-2 JN.1, **F)** SARS-CoV-2 KP.3, **G)** SARS-CoV-1, **H)** RsSHC014, **I)** BtKY72, **J)** PRD-0038, or **K)** Khosta1 S. At least two biological replicates were conducted each in technical duplicates using distinct batches of pseudovirus.

bioRxiv preprint doi: https://doi.org/10.1101/2025.05.11.652904; this version posted May 13, 2025. The copyright holder for this preprint (which was not certified by peer review) is the author/funder, who has granted bioRxiv a license to display the preprint in perpetuity. It is made available under aCC-BY 4.0 International license.

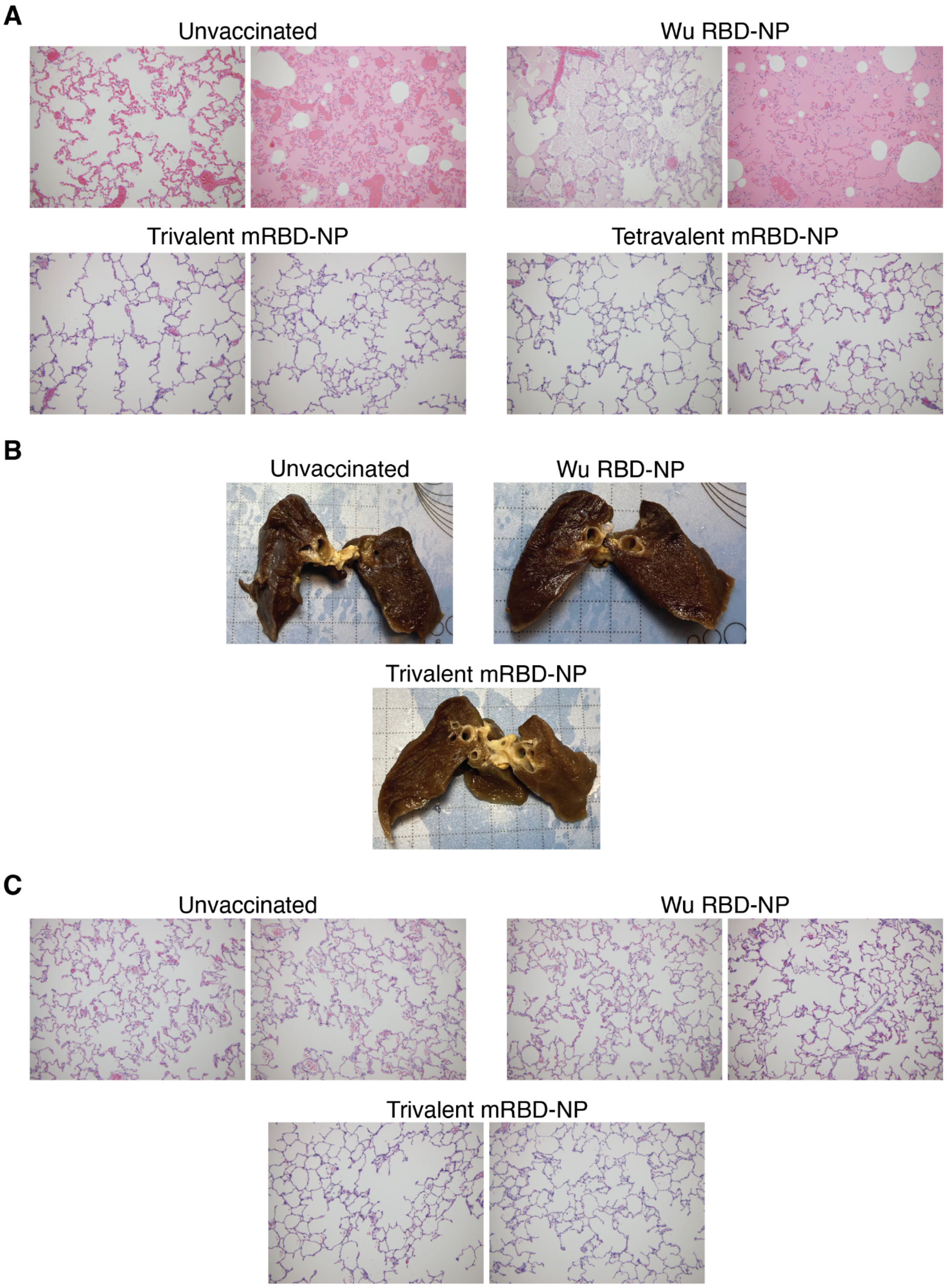

Figure S6. Histological analysis of lungs from SARS-CoV-2 XBB.1.5 and RsSHC014-challenged AGMs. A) Representative images of H&E stained lung tissue from SARS-CoV-2 XBB.1.5-challenged AGMs. **B-C)** Representative images of (B) gross lungs and (C) H&E stained lung tissue from RsSHC014-challenged AGMs.

bioRxiv preprint doi: https://doi.org/10.1101/2025.05.11.652904; this version posted May 13, 2025. The copyright holder for this preprint (which was not certified by peer review) is the author/funder, who has granted bioRxiv a license to display the preprint in perpetuity. It is made available under aCC-BY 4.0 International license.

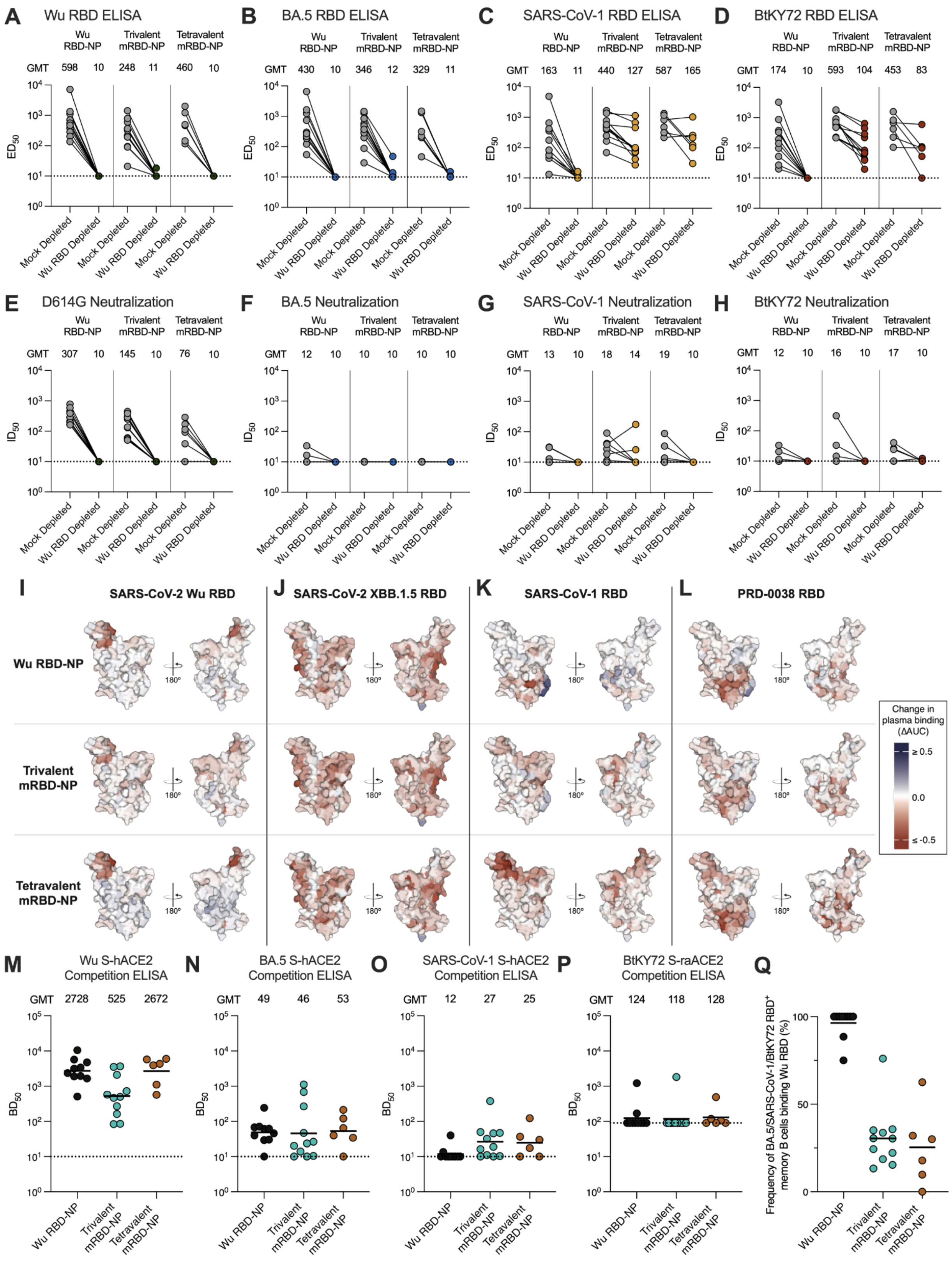

Figure S7. Immune imprinting following one dose of RBD NP in pre-immune AGMs. A-D) Plasma binding titers against the (A) SARS-CoV-2 Wu, (B) SARS-CoV-2 BA.5, (C) SARS-CoV-

bioRxiv preprint doi: https://doi.org/10.1101/2025.05.11.652904; this version posted May 13, 2025. The copyright holder for this preprint (which was not certified by peer review) is the author/funder, who has granted bioRxiv a license to display the preprint in perpetuity. It is made available under aCC-BY 4.0 International license.

1, or (D) BtKY72 RBDs following incubation with uncoated magnetic beads (mock depletion) or Wu RBD-coated magnetic beads (Wu RBD depleted). **E-H)** Plasma neutralizing antibody titers upon mock depletion or Wu RBD depletion against VSV pseudotyped with the (E) SARS-CoV-2 Wu-G614, (F) SARS-CoV-2 BA.5, (G) SARS-CoV-1, or (H) BtKY72 S glycoproteins. Data presented reflect results obtained from one biological replicate with ELISAs and neutralization assays being conducted in technical duplicate and are representative of results obtained from two biological replicates. The geometric mean titers (GMT) are displayed above the plots. The limit of detection (ED_{50}: 1/10 or ID_{50}: 1/10) is represented by a dotted line. **I-L)** Escape mutations mapped for the SARS-CoV-2 Wu (I), SARS-CoV-2 Wu XBB.1.5 (J), SARS-CoV-1 (K), or PRD-0038 (L) RBDs using plasma collected after one dose of the indicated RBD-NPs. Data reflect averaged results from plasma collected from three AGMs with the highest ID50 values against XBB.1.5 in each group. Mutations at sites that increase plasma binding are represented in blue, while those that decrease plasma binding are represented in red. **M-P)** Plasma ACE2 blocking titers using the SARS-CoV-2 (M) Wu or (N) BA.5, (O) SARS-CoV-1, or (P) BtKY72 S glycoprotein. Each data point represents the BD_{50} for a given animal obtained by averaging two biological replicates (independently produced batches of proteins) conducted in technical duplicate. The limit of detection (BD_{50}: 1/10 for Wu, BA.5, and SARS-CoV-1 and 1/90 for BtKY72) is represented by a dotted line. The line indicates geometric mean titers (GMTs). **Q)** Frequency of memory B cells binding to the BA.5/SARS-CoV-1/BtKY72 RBD pool that additionally recognize the Wu RBD in peripheral blood collected after one dose of NP as enumerated by flow cytometry.

bioRxiv preprint doi: https://doi.org/10.1101/2025.05.11.652904; this version posted May 13, 2025. The copyright holder for this preprint (which was not certified by peer review) is the author/funder, who has granted bioRxiv a license to display the preprint in perpetuity. It is made available under aCC-BY 4.0 International license.

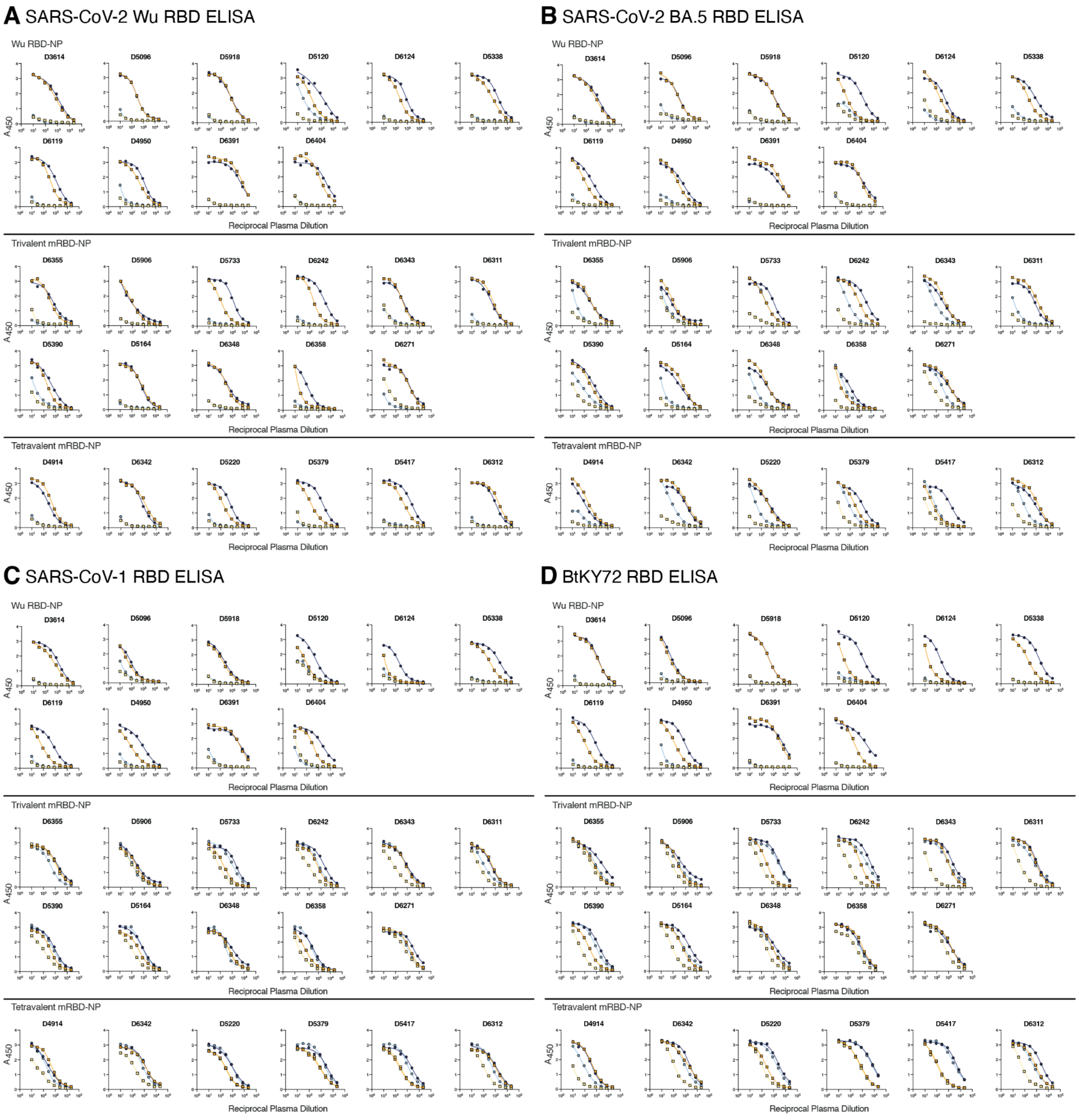

bioRxiv preprint doi: https://doi.org/10.1101/2025.05.11.652904; this version posted May 13, 2025. The copyright holder for this preprint (which was not certified by peer review) is the author/funder, who has granted bioRxiv a license to display the preprint in perpetuity. It is made available under aCC-BY 4.0 International license.

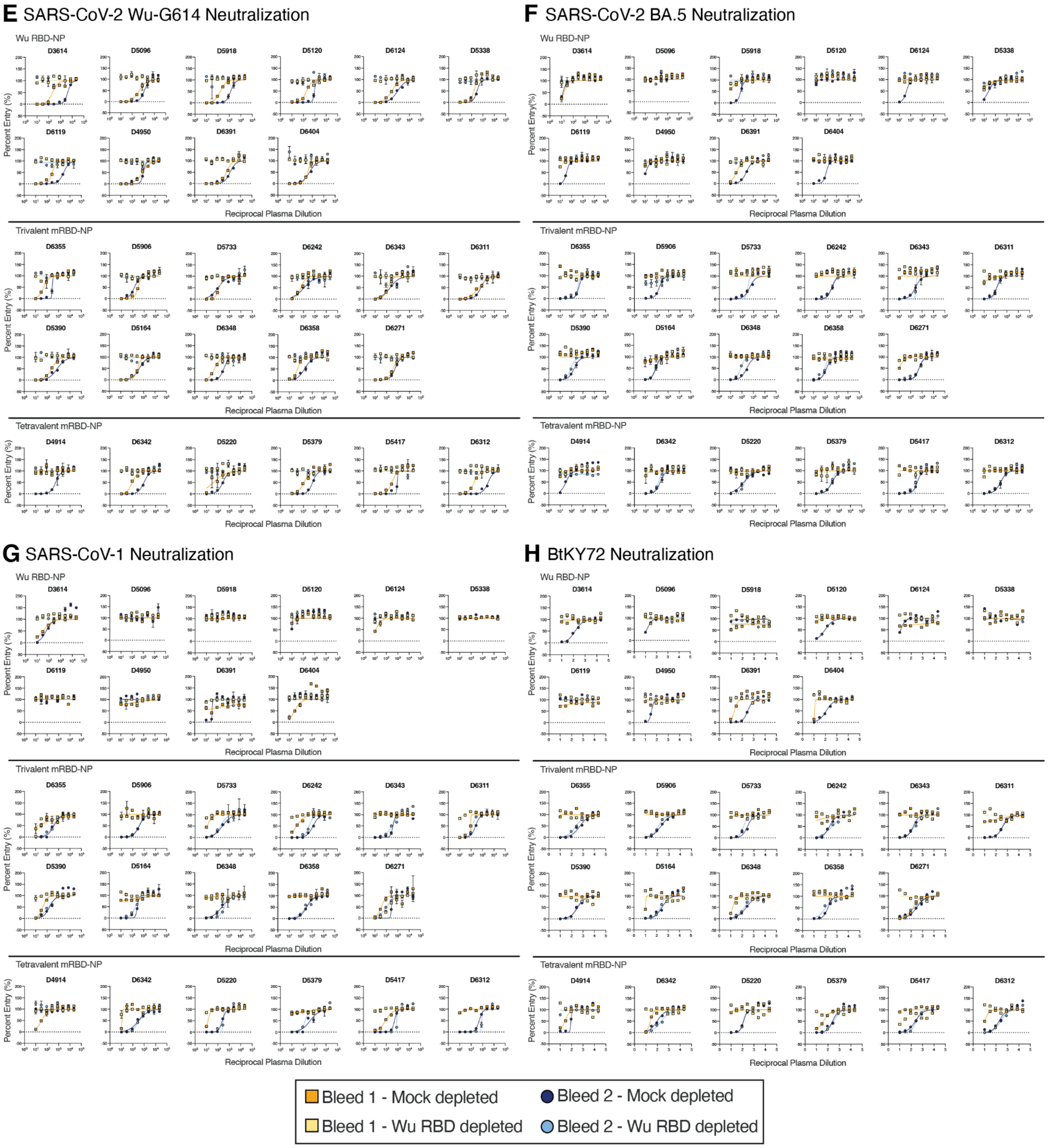

Figure S8. RBD ELISA AND neutralization dose-response curves for RBD-NP-immunized AGMs following mock depletion or depletion of Wu RBD-directed antibodies. Binding antibody titers for mock or Wu RBD depleted plasma were assessed using the **A)** SARS-CoV-2 Wu, **B)** SARS-CoV-2 BA.5, **C)** SARS-CoV-1, or **D)** BtKY72 RBD. Neutralizing antibody titers for mock or Wu RBD depleted plasma were assessed using VSV pseudotyped with the **E)** SARS-

bioRxiv preprint doi: https://doi.org/10.1101/2025.05.11.652904; this version posted May 13, 2025. The copyright holder for this preprint (which was not certified by peer review) is the author/funder, who has granted bioRxiv a license to display the preprint in perpetuity. It is made available under aCC-BY 4.0 International license.

CoV-2 Wu, **F)** SARS-CoV-2 BA.5, **G)** SARS-CoV-1, or **H)** BtKY72 S. Data presented are from one biological replicate with both ELISAs and neutralization assays conducted in technical duplicate and representative of results from a second independent depletion experiment completed with a distinct batch of proteins and pseudoviruses.

bioRxiv preprint doi: https://doi.org/10.1101/2025.05.11.652904; this version posted May 13, 2025. The copyright holder for this preprint (which was not certified by peer review) is the author/funder, who has granted bioRxiv a license to display the preprint in perpetuity. It is made available under aCC-BY 4.0 International license.

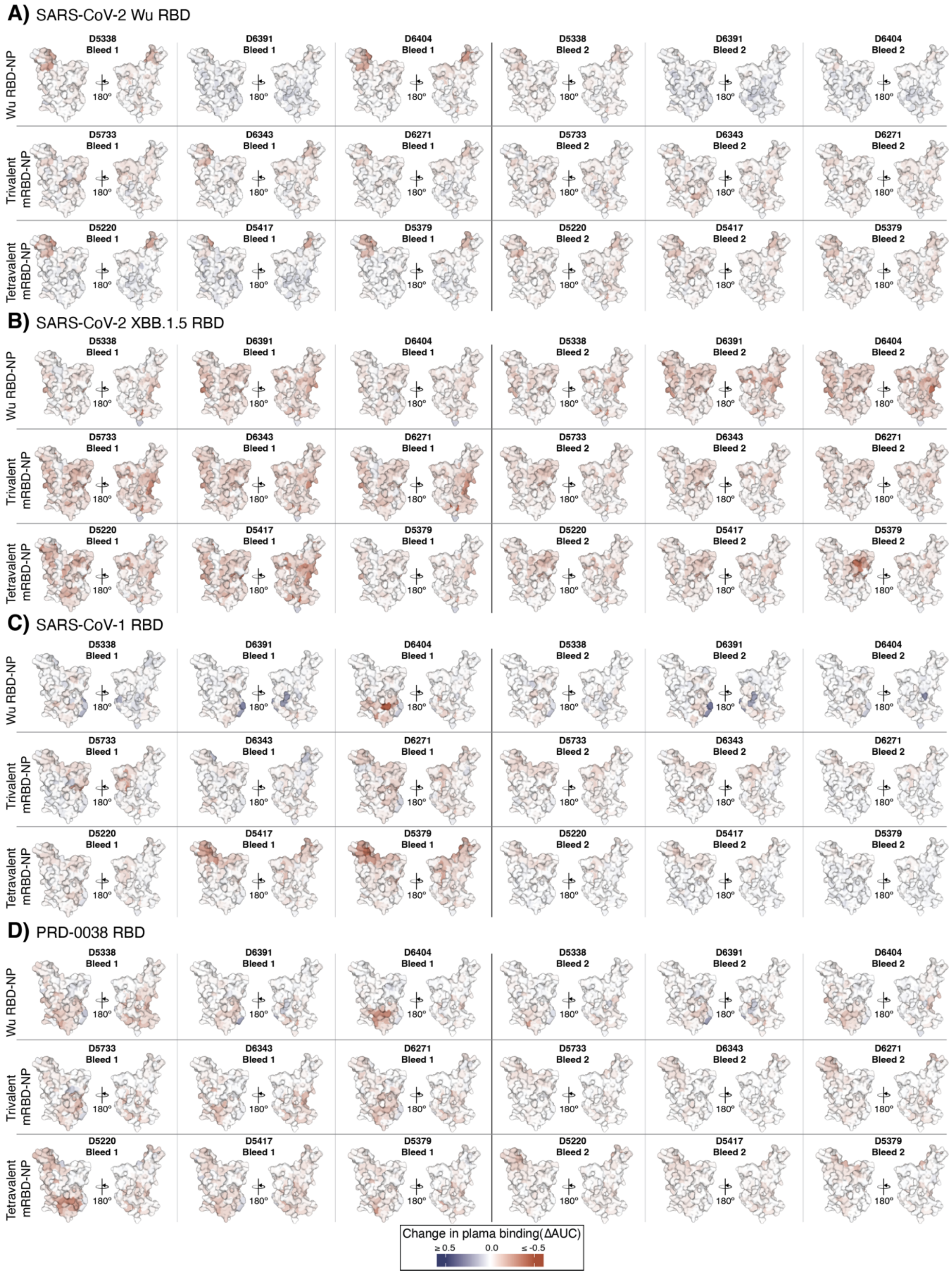

Figure S9. Deep mutational scanning data for RBD escape mutations for individual AGMs. Plasma escape mutations mapped for each of the 9 AGMs profiled using the **A)** SARS-CoV-2 Wu, **B)** SARS-CoV-2 XBB.1.5, **C)** SARS-CoV-1, or **D)** PRD-0038 RBD DMS libraries.

bioRxiv preprint doi: https://doi.org/10.1101/2025.05.11.652904; this version posted May 13, 2025. The copyright holder for this preprint (which was not certified by peer review) is the author/funder, who has granted bioRxiv a license to display the preprint in perpetuity. It is made available under aCC-BY 4.0 International license.

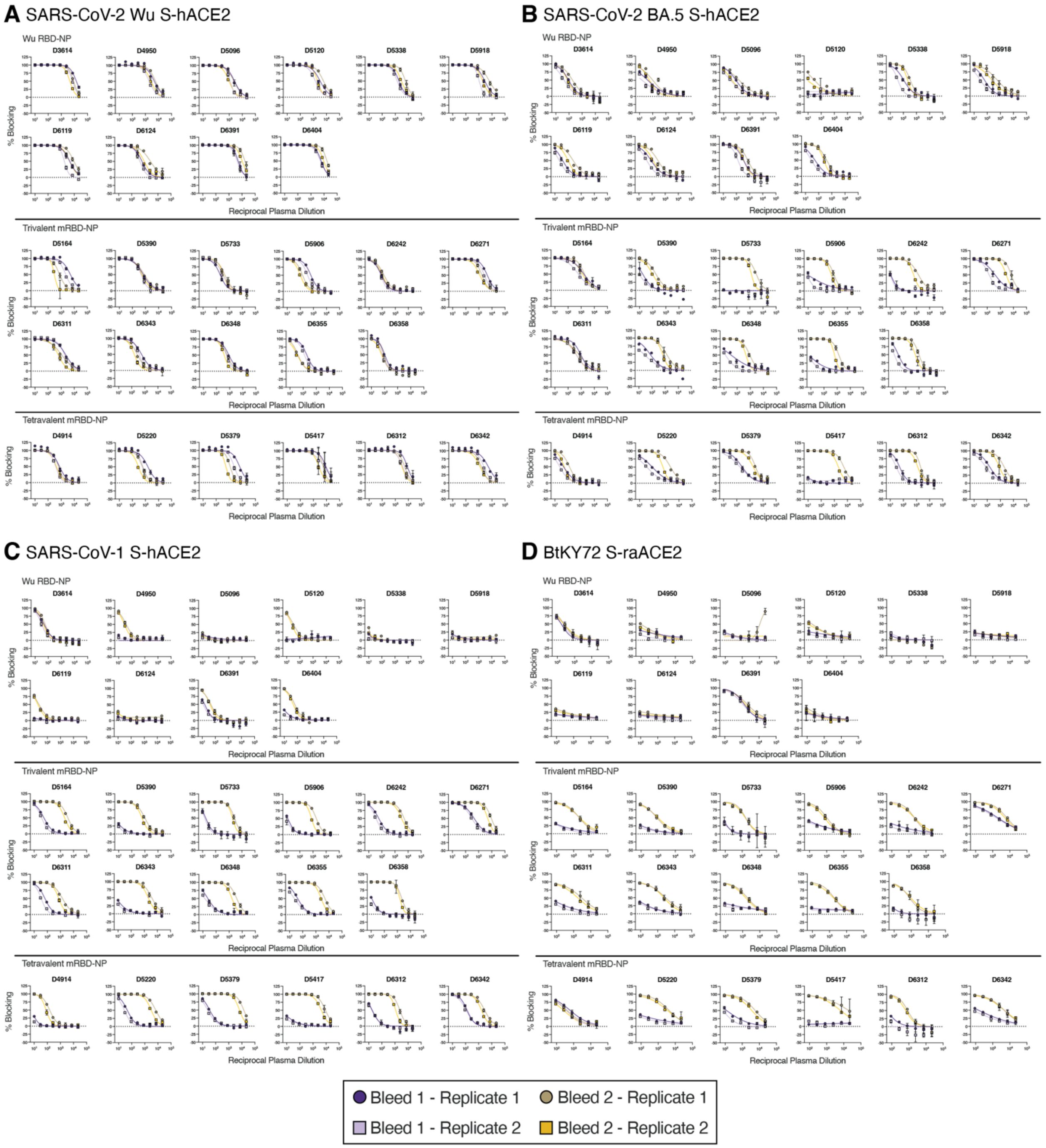

Figure S10. Human or *R. alcyone* ACE2 competition ELISA dose-response curves for RBD-NP-immunized AGMs. Plasma ACE2 blocking titers were assessed using the **A)** SARS-CoV-2 Wu, **B)** SARS-CoV-2 BA.5, or **C)** SARS-CoV-1 S and dimeric human ACE2 (hACE2) or **D)** BtKY72 S and dimeric *R. alcyone* ACE2 (raACE2). Two biological replicates were each conducted in technical duplicates using distinct batches of proteins.

bioRxiv preprint doi: https://doi.org/10.1101/2025.05.11.652904; this version posted May 13, 2025. The copyright holder for this preprint (which was not certified by peer review) is the author/funder, who has granted bioRxiv a license to display the preprint in perpetuity. It is made available under aCC-BY 4.0 International license.

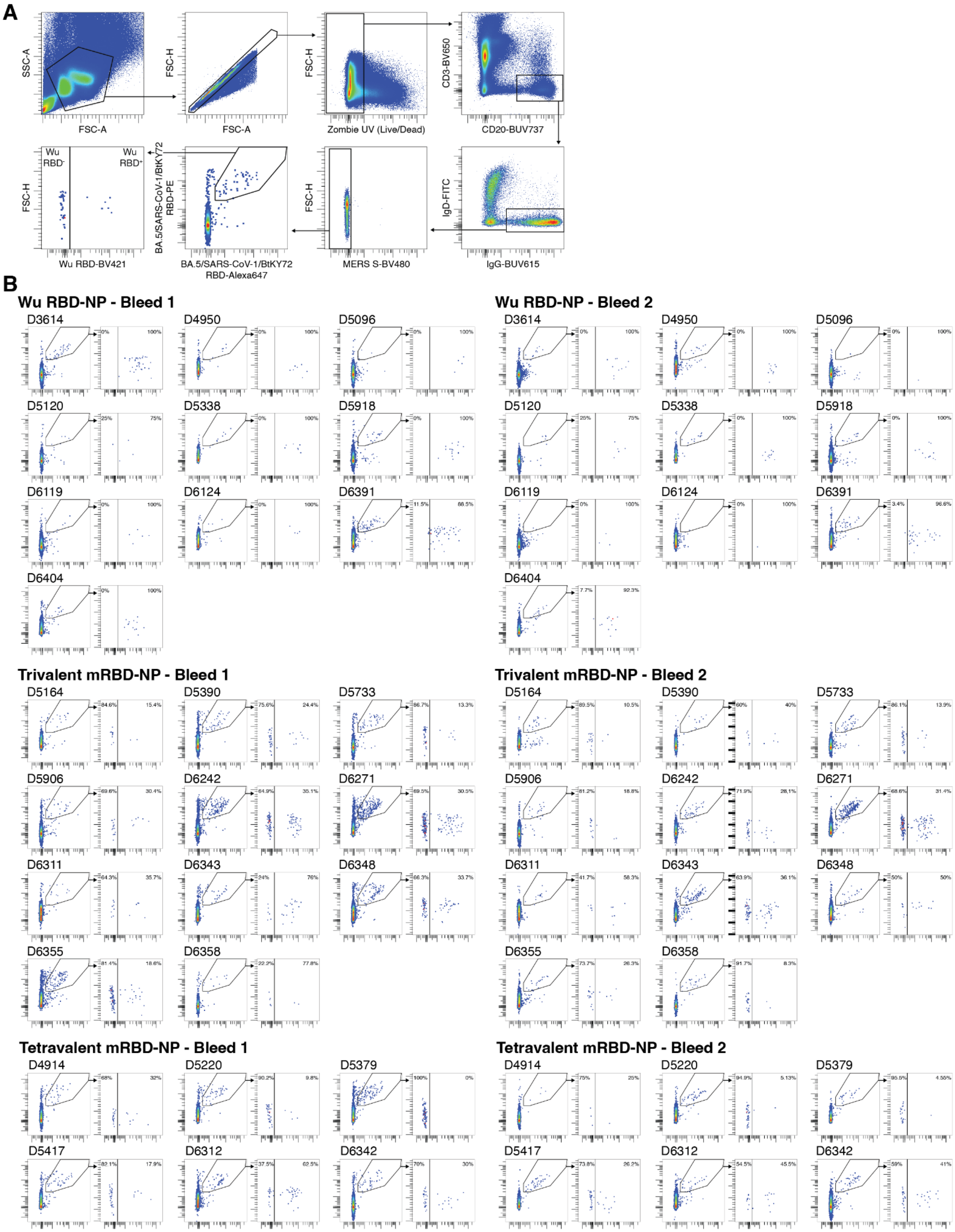

Figure S11. Flow cytometry analysis of memory B cells collected from RBD-NP-immunized AGMs. A) Gating strategy to determine binding specificity of memory B cells in

bioRxiv preprint doi: https://doi.org/10.1101/2025.05.11.652904; this version posted May 13, 2025. The copyright holder for this preprint (which was not certified by peer review) is the author/funder, who has granted bioRxiv a license to display the preprint in perpetuity. It is made available under aCC-BY 4.0 International license.

peripheral blood collected from RBD-NP-immunized AGMs. **B)** Evaluation of RBD-reactivity of memory B cells for each individual AGM.
